# Supplementary figures and images for: The Paramecium histone chaperone Spt16-1 is required for Pgm endonuclease function in programmed genome rearrangements
Source: PLoS Genet. 2020 Jul 23;16(7):e1008949. doi: 10.1371/journal.pgen.1008949 (PMC7402521; doi:10.1371/journal.pgen.1008949)

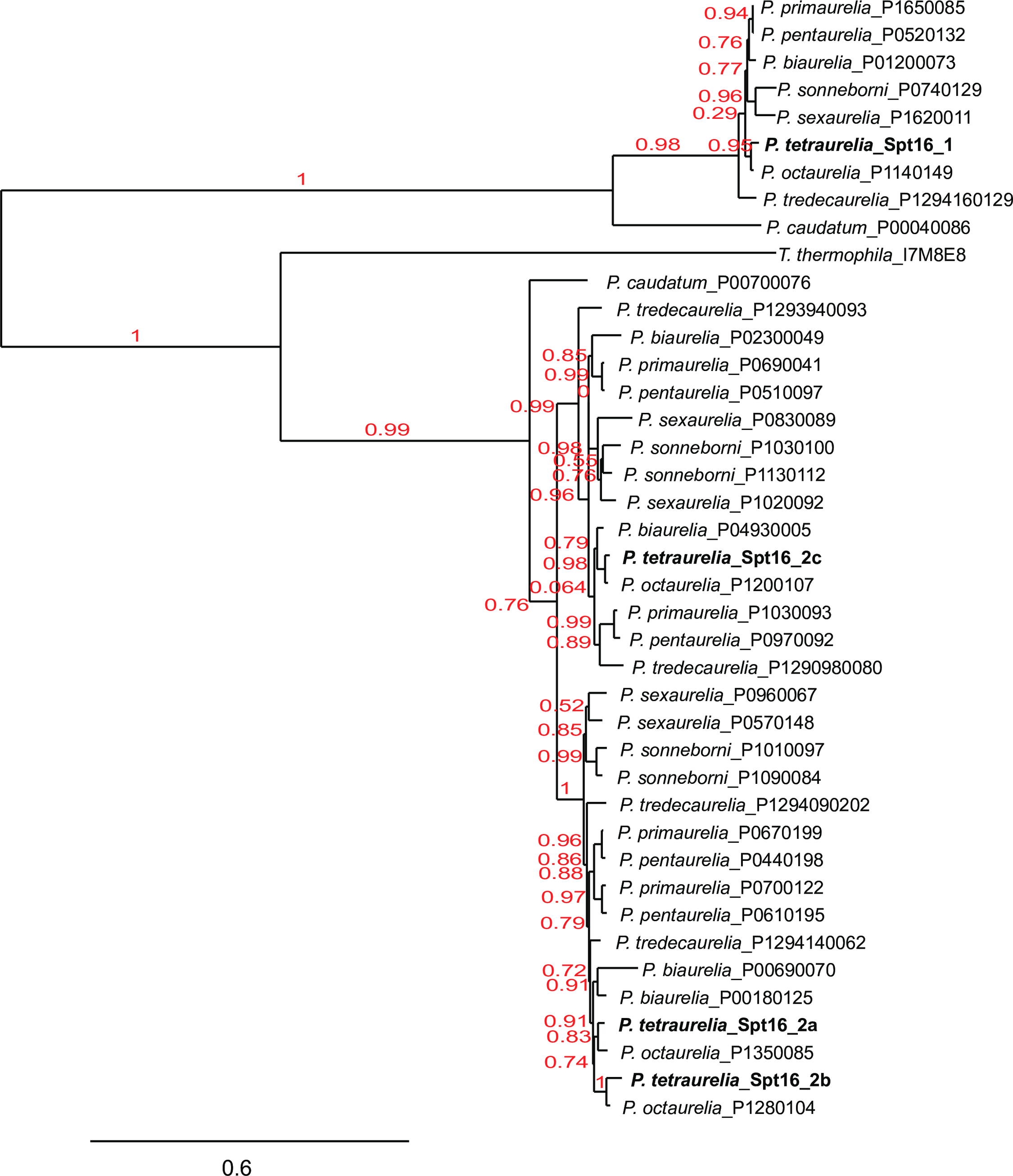

Supplement: S1 Fig — Phylogenetic tree of Spt16 proteins from P. tetraurelia, other sequenced Paramecium species and other eukaryotes based on alignment of full length protein sequence with MUSCLE. Phylogeny of the alignment was generated using PhyML 3.0 on Phylogeny.fr with bootstrapping procedure using 100 bootstraps. Tree has been viewed using Tree.Dyn 198.3. Accession numbers are indicated in S1 Table. (TIF) [file pgen.1008949.s001.tif]

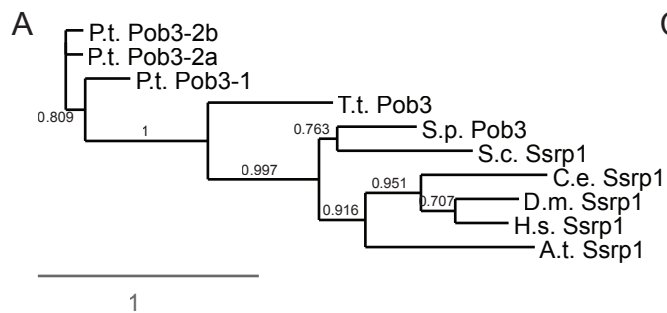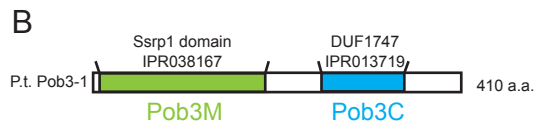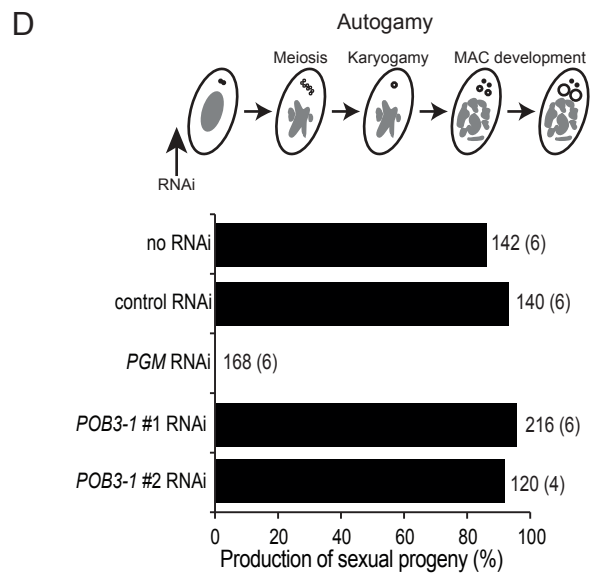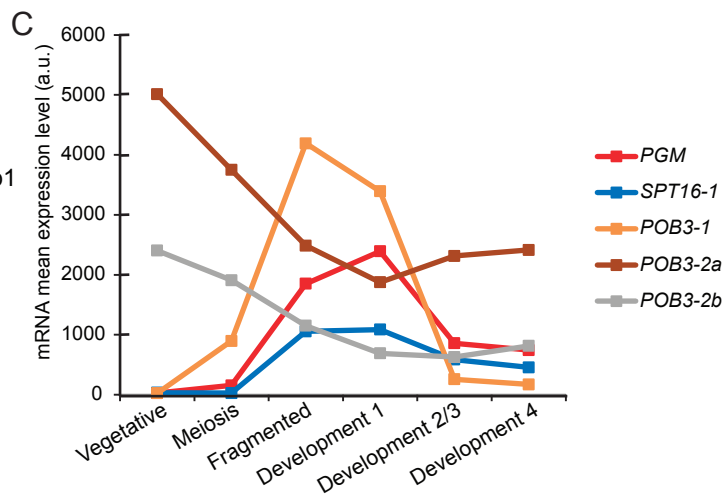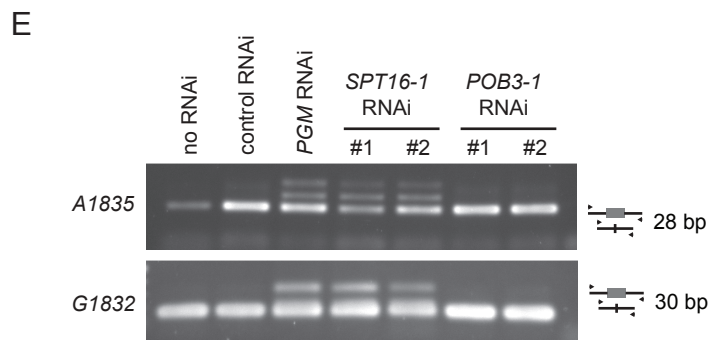

Supplement: S3 Fig — (A) Phylogenetic tree of Pob3 proteins from Paramecium tetraurelia (Pt), Tetrahymena thermophila (Tt), Saccharomyces cerevisiae (Sc), Schizosaccharomyces pombe (Sp), Caenorhabditis elegans (Ce), Drosophila melanogaster (Dm), human (Hs) and Arabidopsis thaliana (At) based on the alignment of full-length protein sequences. The tree was generated with PhyML 3.0 with bootstrapping procedure and visualized with Tree.Dyn 198.3. Accession numbers are provided in S1 Table. (B) Conserved domains (colored boxes) in P. tetraurelia Pob3-1 protein. (C) POB3 gene expression profiles during the life cycle. Mean mRNA expression levels were determined by RNA sequencing during vegetative growth and at different time points during autogamy [31]. (D) Production of post-autogamous sexual progeny following POB3-1 gene silencing. The gene targeted in each silencing experiment is indicated. Two non-overlapping silencing fragments (#1: positions 36–420 and #2: positions 442–878 of PTET.51.1.G0610231) of the POB3-1 gene were used independently. The ND7 or ICL7 genes were used as control RNAi targets, since their silencing has no effect on sexual processes [21]. The total number of autogamous cells analyzed for each RNAi and the number of independent experiments (in parenthesis) are indicated. The absence of lethality observed after POB3-1 KDs should be taken with caution as the level of KDs was not measured. (E) PCR analysis of IES retention. Primers (black arrows, S2 Table) are located on either side of the IES. Total DNA samples were prepared from autogamous cell population upon RNAi-mediated silencing of the indicated genes. Because the maternal MAC is still present at this stage, the excised version is amplified in all cases; the IES-retaining fragment can be detected only if it accumulates in the zygotic developing MACs. (PDF) [file pgen.1008949.s003.pdf]

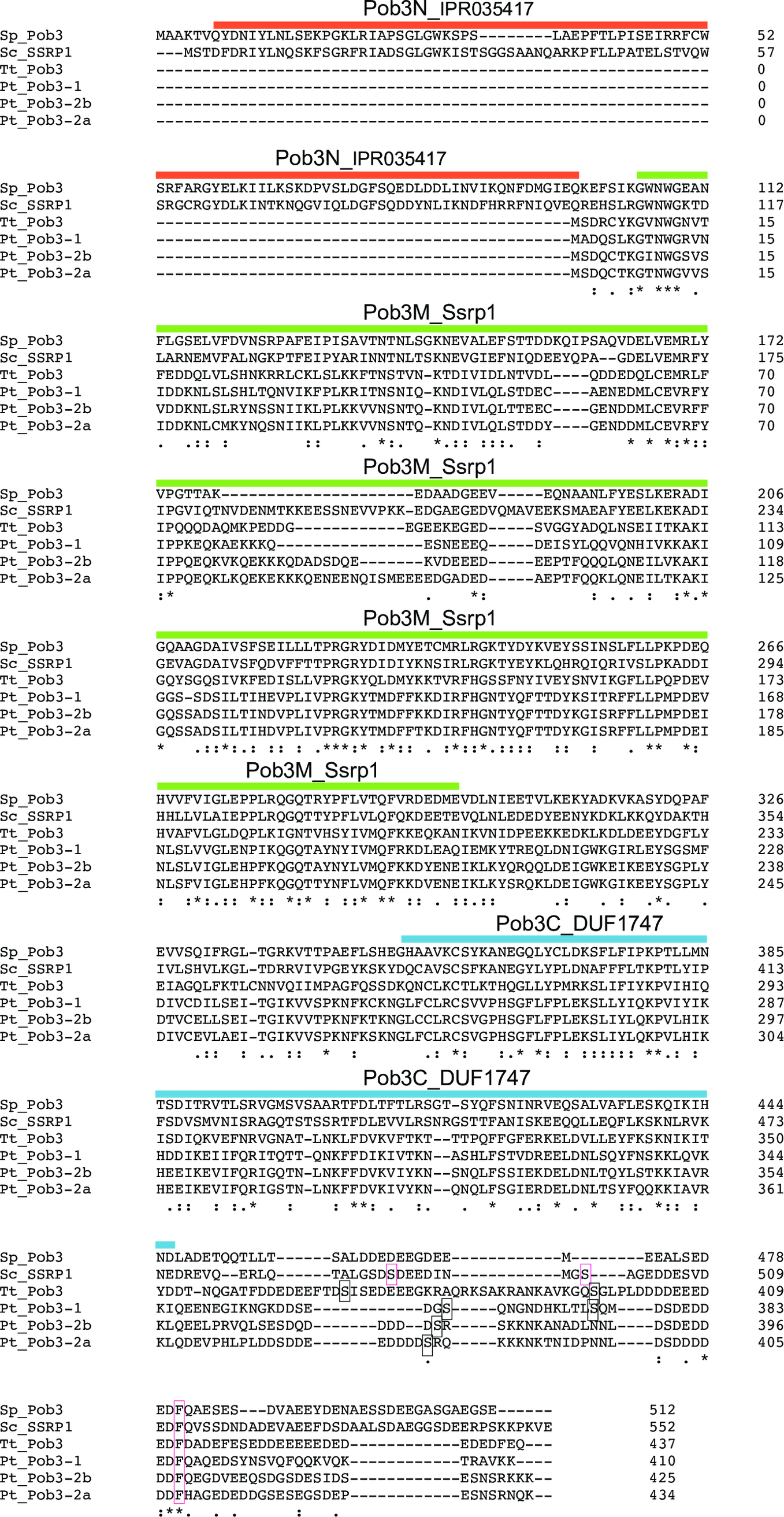

Supplement: S4 Fig — CLUSTAL Omega alignment of Pob3 homologues from P. tetraurelia and other eukaryotes. Domains identified by Interpro are indicated in colors. The Pob3M and Pob3C domains contain two PH motifs, similar to SPT16 M domain, which are likely to be involved in binding H3/H4 due to the strong similarity with the dual PH motifs of Rtt106, a known H3/H4 chaperone. Paramecium and Tetrahymena Pob3 proteins lack the Pob3_N domain. The Pob3_N domain is required for dimerization with SPT16. Residues important for interaction of Sc_SSRP1-CTD with H2A-H2B dimers are shown with pink squares [52]. P.t. Paramecium tetraurelia, T.t Tetrahymena thermophila, S.p. Schyzosaccharomyces pombe, S.c. Saccharomyces cerevisiae. (TIF) [file pgen.1008949.s004.tif]

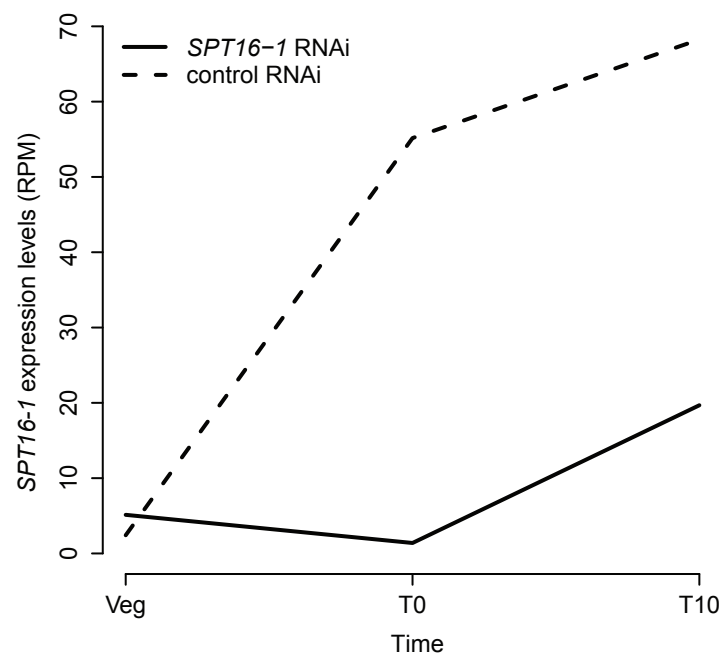

Supplement: S5 Fig — The expression levels of SPT16-1 mRNA are measured using normalized read counts (RPM, Reads per million mapped reads) at three time points (Vegetative (Veg), T0 and T10) during autogamy upon SPT16-1 (solid line) and control (dashed line) RNAi. Only the nucleotides outside of the targeted RNAi region within the SPT16-1 gene are considered. (PDF) [file pgen.1008949.s005.pdf]

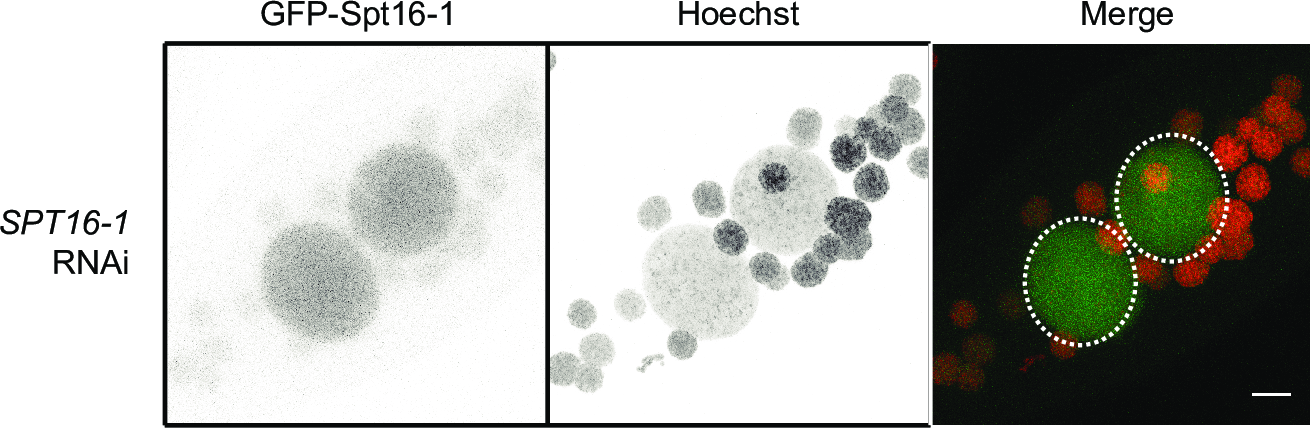

Supplement: S6 Fig — Overlay of Z-projections of magnified views of GFP-Spt16-1 (green) and Hoechst (red) in SPT16-1 RNAi during MAC development. Dashed white circles indicate the two developing MACs. The other Hoechst-stained nuclei are fragments from the maternal somatic MAC. Scale bar is 10 μm. (TIF) [file pgen.1008949.s006.tif]

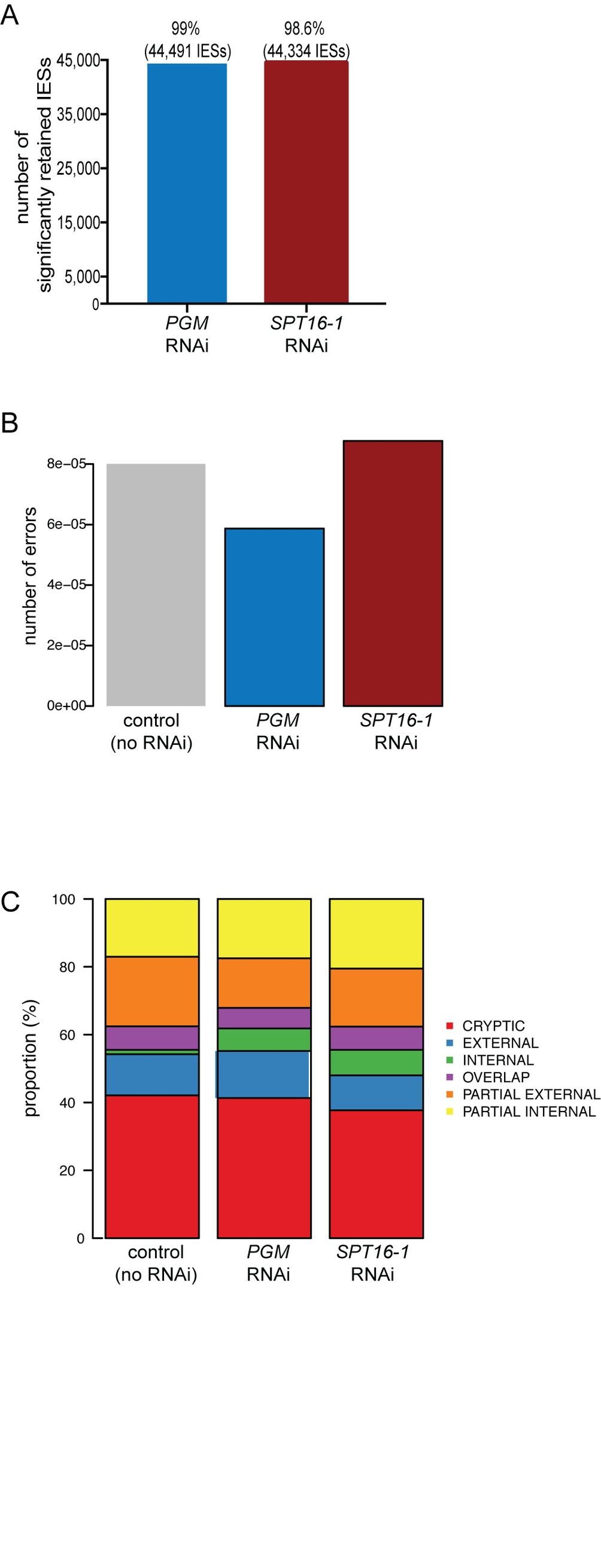

Supplement: S7 Fig — (A) Excision of all IESs is altered upon SPT16-1 KD. 98.6% (44,334) IESs are statistically significantly retained in the developing MAC after SPT16-1 KD, similar to the 99% (44,491) observed after PGM KD (sample from [12)]). (B) Quantification of excision errors between control (no RNAi), SPT16-1 and PGM RNAi (FACS-sample from [11]). (C) Quantification and identification of error types are made with the PARTIES software described in [36]. There is no significant difference between the three conditions. (TIF) [file pgen.1008949.s007.tif]

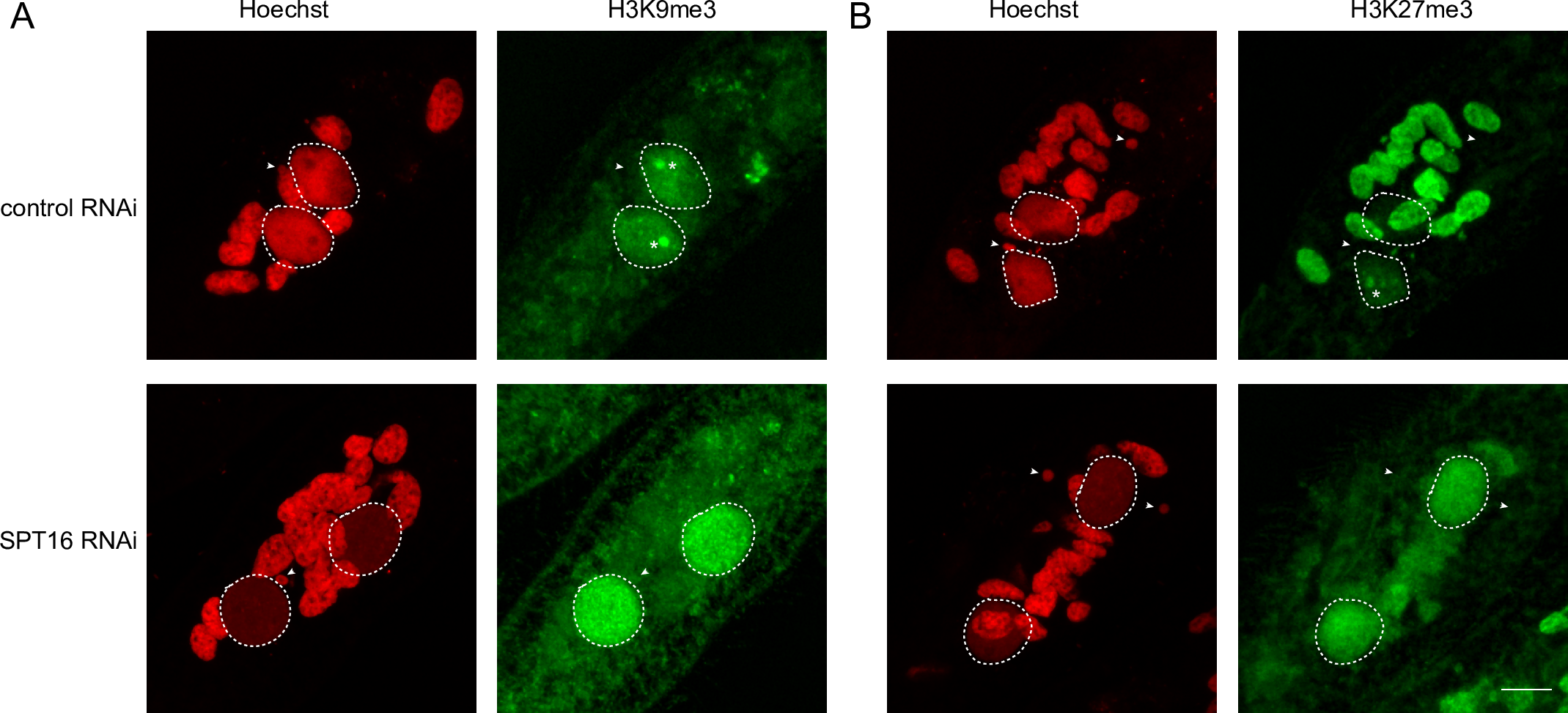

Supplement: S8 Fig — Z-projections of immunolabelling with (A) H3K9me3-specific antibodies (green) or (B) H3K27me3-specific antibodies and staining with Hoechst (red) in control or SPT16-1 RNAi at late stages of development. Dashed white circles indicate the two developing MACs. White arrowheads indicate the MICs. The other Hoechst-stained nuclei are fragments from the maternal somatic MAC. Scale bar is 10 μm. (TIF) [file pgen.1008949.s008.tif]

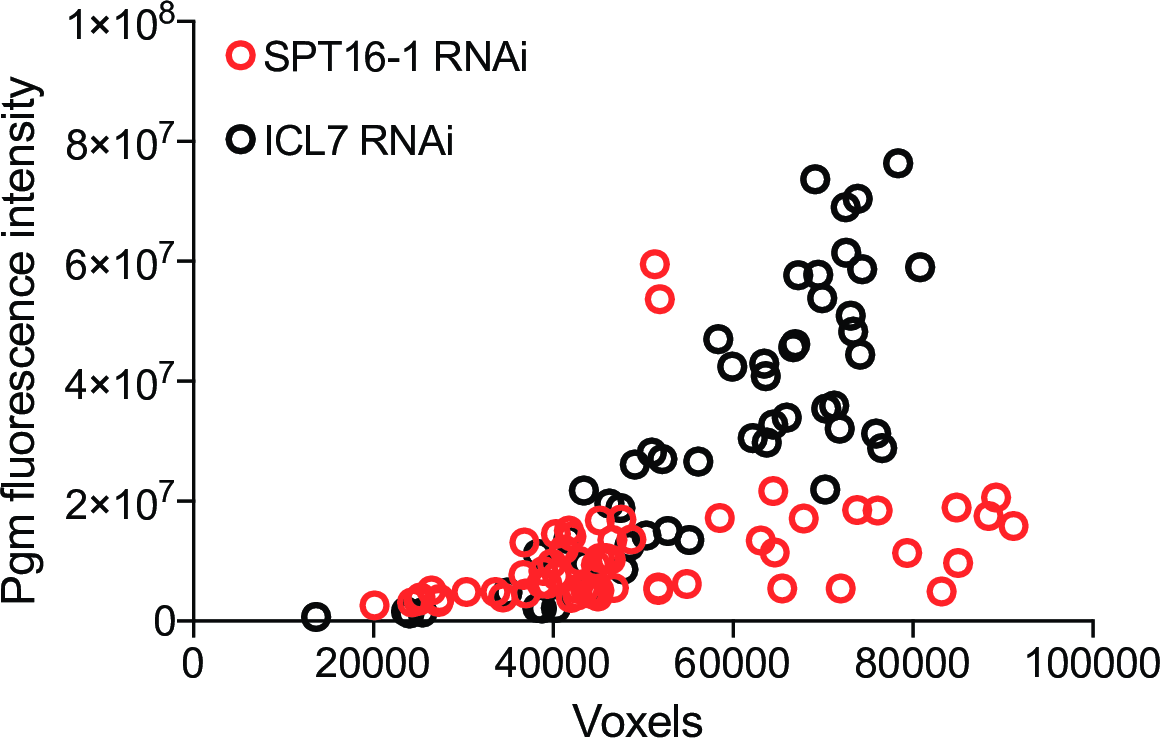

Supplement: S9 Fig — The Pgm fluorescence intensity is plotted as a function of the estimated nucleus volume in voxels in control or SPT16-1 KD cells (same data as Fig 7A). The estimated volume of the developing macronucleus increases as development progresses in control and SPT16-1 KD cells. (TIF) [file pgen.1008949.s009.tif]
