# Supplementary material for: The Paramecium histone chaperone Spt16-1 is required for Pgm endonuclease function in programmed genome rearrangements
Source: PLoS Genet. 2020 Jul 23;16(7):e1008949. doi: 10.1371/journal.pgen.1008949 (PMC7402521; doi:10.1371/journal.pgen.1008949)
Supplement: S2 Fig — MUSCLE alignment of Spt16 homologues from P. tetraurelia and other eukaryotes. Domains idientified by Interpro are indicated in colors. Conserved aromatic and acidic residues in MBD are shown with asterisk (*). Conserved residues are highlighted in black and grey (amino acids with same physical and chemical properties). P.t. Paramecium tetraurelia, T.t Tetrahymena thermophila, S.p. Schyzosaccharomyces pombe, S.c. Saccharomyces cerevisiae, C.e. Caenorhabditis elegans, D.m. Drosophila melanogaster, H.s. Homo sapiens, M.m. Mus musculus, A.t. Arabidopsis thaliana. (PDF) [file pgen.1008949.s002.pdf]

Spt16N_N Lobe

```
P.t._SPT16-1    1 --------------------MDKVQIALQNKFKKNNKLAITTLRK--------ANQFDCLAVLIGTSHTSNIGIQ-KGFQ
T.t._SPT16      1 ----------------------MSKVKADI-FYQHHKQLLSVWNK-----QPQYANIDAFIIKNGK-DQGGNKIKTSAIS
P.t._SPT16-2c   1 ---------------------MDKKIIADV-FQRHLDQLTSRLND----------DLPALCILSGK-EDGSIKPKTKALF
P.t._SPT16-2a   1 ---------------------MDKKIIADV-FQRHLEQLVSRLNE----------DVPAICILSGK-EDGSIKPKTKALF
P.t._SPT16-2b   1 ---------------------MDKKIIADV-FQRHLEQLVSRLND----------DIPAICILSGK-EDGSIKPKTKALF
A.t._SPT16      1 MADSRNGNARAPPSGVPPKAGNTYSIDVKN-FISRARALYEHWKK--HS-ADLWGSADALAIATPPASDDLRYLKSSALN
S.p._Spt16      1 --------------------MAEYEIDEIT-FHKRLGILLTSWKNEEDG-KTLFQDCDSILVTVGAHDDTNPYQKSTALH
S.c._Spt16      1 --------------------MEELNIDFDV-FKKRIELLYSKYNE-------FEGSPNSLLFVLGSSNAENPYQKTTILH
C.e._SPT16      1 ------------------MSGKRAVLNKDL-FFQRAERLYEHWEK---G-ADGLDSIKSLAFVYG--ETDNPYTKTSALF
D.m._Dre4       1 --------------------MSSFVLDKEA-FVRRVKRLYTEWRAPSIGHDDALRNLDCIMSIVGV-EEDVMYSKSMALQ
H.s._SPT16H     1 ---------------------MAVTLDKDA-YYRRVKRLYSNWRK---G-EDEYANVDAIVVSVGV-DEEIVYAKSTALQ
M.m._Supt16     1 ---------------------MAVTLDKDA-YYRRVKRLYSNWRK---G-EDEYASIDAIVVSVGV-DEEIVYAKSTALQ
```

Spt16N_N Lobe

```
P.t._SPT16-1   52 QWYLGCELMDCILIMSTKMLCIIADEVMFQKLKHL-----SDIKMKTFTIFFLIKNIKKNNH---QQFQFALERLRKEYP
T.t._SPT16     52 MWYFGFDFIDTILLITKKTFAIIGGNKKINMLKSVQ----EHAEAKEYNLVFIEKDQANNSNQLQQLFEILDKDLNKSS-
P.t._SPT16-2c  48 VWLFGYDMIETVFIATRKQIFYLASDKKLQMMEET-----KQRLGGRYEVHFYKKVADNRDS-----FEKIRQKLGN---
P.t._SPT16-2a  48 VWLFGYDMIETVFLATKKQIFYLASDKKLQMMEET-----KQKLSGKFEVHFYKKGNDNRES-----FDKIRQKLGN---
P.t._SPT16-2b  48 VWLFGYDMIETVFLATKKQIFYLASDKKLQMMEET-----KHKLSGKFEVHFYKKMNDNKDA-----FDKIRQKIGN---
A.t._SPT16     77 IWLLGYEFPDTIMVFTKKQIHFLCSRNKASLLEVV-----KKPAHDELKLDVIMHVKPKGDD-GTGLMDAIFRAIRDLSR
S.p._Spt16     59 TWLLGYEFPSTLILLEKHRITILTSVNKANMLTKL-----AETKGAAADVNILKRTKDAEEN--KKLFEKIIEYIRATN-
S.c._Spt16     53 NWLLSYEFPATLIALVPGKVIIITSSAKAKHLQKAIDL--FKDPESKITLELWQRNNKEPEL-NKKLFDDVIALINSAG-
C.e._SPT16     56 TWLFGHEIADTVLLLLKDHIYILGSNRKVEFFGSVTGD--NQSSGKVPTVSTLLRDKTDKDA---GNFEKLIDHIKSAG-
D.m._Dre4      59 LWLLGYELTDTISVFCSDAVYFLTSKKKIEFLKQTQ----NITEEGFPEINLLVRDRTDKDQ---GNFEKLIKALQNSKK
H.s._SPT16H    54 TWLFGYELTDTIMVFCDDKIIFMASKKKVEFLKQIANTKGNENANGAPAITLLIREKNESNK---SSFDKMIEAIKESKN
M.m._Supt16    54 TWLFGYELTDTIMVFCDDKIIFMASKKKVEFLKQIANTKGNENANGAPAITLLVREKNESNK---SSFDKMIDAIKESKS
```

Spt16_N Lobe | Spt16N_Peptidase M24

```
P.t._SPT16-1  124 G---NNYRLALNLSDGQKSPLITEFNQFID----QNHLIKVDCTSFLKELINNDNKDIFEYYNTCGKINSYYMKF-MSQR
T.t._SPT16    127 ------FNIGTLAKEQQVGPFMTEYDSFIKD---KNQYKFADCSVFVQDCLSVKDQNEISYIGKAAKVSVYLESK-LIKE
P.t._SPT16-2c 115 ------GKLGMPTTEQQAGSLATEWYDY------KGWSQIVDASQLISDVLAVKDDQEQAFINQSSQLTTRLFKK-LIKQ
P.t._SPT16-2a 115 ------VKLGMPTTEKQAGSLAAEWYEY------KGWQQIVDANQLISDVLAVKDDQEQGFIQQSSQLTTRLFKK-LIKQ
P.t._SPT16-2b 115 ------VKLGMPTTEKQAGSLAAEWYEY------KGWQQIVDATQLIGDVLAVKDDQEQGFIQQSSQLTTRLFKK-LIKQ
A.t._SPT16    151 GDGNDSQVVGHIAREAPEGKLLETWTERLK----NANFQFVDITGGLSDLFAVKDDTEVMSVKKAAYLAYSVMKNVVVPN
S.p._Spt16    131 ------KKVGVFPKDKTQGKFINEWDSIFEPV--KSEFNLVDASLGLAKCLAIKDEQELANIKGASRVSVAVMSKYFVDE
S.c._Spt16    129 ------KTVGIPEKDSYQGKFMTEWNPVWEAAVKENEFNVIDISLGLSKVWEVKDVNEQAFLSVSSKGSDKFMDL-LSNE
C.e._SPT16    130 ------GDVGNFVKEKFSSEFVSSWNKALE----EGGVNKNDVTLAFTHLFAVKDDKEMDLIRKSAQATTASWTA-ARAR
D.m._Dre4     132 G-----KRLGVFAKDAYPGEFSEAWKKSLT----ASKFEHVDISTIIAYLMCPKDESEINNIRKASLVSMDIFNKYLKDE
H.s._SPT16H   131 G-----KKIGVFSKDKFPGEFMKSWNDCLN----KEGFDKIDISAVVAYTIAVKEDGELNLMKKAASITSEVFNKFFKER
M.m._Supt16   131 G-----KKIGVFSKDKFPGEFMKSWSDCLN----KEGFDKVDISAVVAYTIAVKEDGELNLMKKAASITSEVFNKFFKER
```

Spt16N_Peptidase M24

```
P.t._SPT16-1  196 IELAIKFNENTTNYSITQAVKREKSSDLNQMAIRRKFGL--------Q-GNYDILS-STVQSGGQYNVSASE-STQSRLV
T.t._SPT16    197 IETIIEDEGKKTHSQIATMIEGLIENEKELKKISEEIGG--------ESDNLDLAYVPIVQSGGKYDLKPNAQSNEDILS
P.t._SPT16-2c 182 IEDSIDVGTRITHQDLAKKVEQSLDNDKQ--KVIKDIGL--------QDGLYDFAYTPIIQSGGNY--QQVDGPNKDYLS
P.t._SPT16-2a 182 IEDSIDVGTRITHQDLAKKVEQSLENDKQ--KVMKEIGL--------QDGLYDFAYTPIIQSGGNY--QQVDGPNKDYLS
P.t._SPT16-2b 182 IEDSIDVGTRITHQDLAKKVEQSLENDKQ--KVMKEIGL--------QDGLYDFAYTPIIQSGGNY--QQVEGPNKDYLS
A.t._SPT16    227 LESAIDEEKDVTHSALMDLTEKAILEPTK-----ASVKL--------KPENVDICYPPIFQSGGKFDLKPSAASNDELLT
S.p._Spt16    203 LSTYIDQGKKITHSKFSDQMESLIDNEAFFQTKSLKLGDI-------DLDQLEWCYTPIIQSGGSYDLKPSAITDDRNLH
S.c._Spt16    202 MVRAVDEELKITNAKLSDKIENKIDDVKFLKQLSPDLSALCPPNYKFNFDLLDWTYSPIIQSGKKFDLRVSARSTNDQLY
C.e._SPT16    199 YVEIIDQEKRVRHSVLSNEFAAFMKDSKV------QQAL--------AKYEADTCYDPIVMSGGNYSFKWNHESSESHLH
D.m._Dre4     203 IMDIIDSDRKVKHNKLSDGCEAAIGEKKY------TSGL--------DPRLLDMAYPPIIQSGGAYSLKFSAVADKNPLH
H.s._SPT16H   202 VMEIVDADEKVRHSKLAESVEKAIEEKKY------LAGA--------DPSTVEMCYPPIIQSGGNYNLKFSVVSDKNHMH
M.m._Supt16   202 VMEIVDADEKVRHSKLAESVEKAIEEKKY------LAGA--------DPSTVEMCYPPIIQSGGNYNLKFSVVSDKNHMH
```

Spt16N_Peptidase M24

```
P.t._SPT16-1    265 GD---VVIYSFCCQYMQSQSYCTRTLLFQPNQELEQIYRVILNVHAFALG-LVKEDIQFKQIYRETQNIWETIFKDDPEM
T.t._SPT16      269 YD---TIIVSVGTKYMEYHANIVRTLFIDPTNDQKKIYQRVYELQNQIAV-QLKPGIKLKTVYENAVNFIN---EKVPQL
P.t._SPT16-2c   250 SD---VIIISLGTQVNEYNTNCIRTLFINPTEVQKKLYNTIIEVQSKIIG-LLTVGTPLNQVYKEAVPILQ---QKIQEI
P.t._SPT16-2a   250 SD---VIIIQLGTQVNEYNTNCIRTLFINPTEIQKKLYNAILEVQSKIIT-LMTIGVSLNVVFKESLQLLQ---YKLQEL
P.t._SPT16-2b   250 SD---VIIIQLGTQVNEYNTNCIRTLFINPTEIQKKLYNAILEVQNKIIT-LMTIGTTLNSVFKEALQLLQ---QKLQEL
A.t._SPT16      294 YDPASIIICAVGARYNSYCSNVARTYLIDATSLQSKAYEVLLKAHEAAID-ALRSGRKINTVYQAALSVVE---KNAPEF
S.p._Spt16      276 GD---VVLCSLGFRYKSYCSNVGRTYLFDPDSEQQKNYSFLVALQKKLFE-YCRDGAVIGDIYTKILGLIR---AKRPDL
S.c._Spt16      282 GN--GCILASCGIRYNNYCSNITRTFLIDPSEEMANNYDFLLTLQKEIVTNILKPGRTPKEVYESVIEYIE---KTKPEL
C.e._SPT16      265 SQ-FGTIITSFGARLSEYCTNLTRTMLIFPSSELETAYEAILAAELAVIA-ALKPGAKLSDVYKIGIDTLT---EKSPKL
D.m._Dre4       269 FG---VIVCSLGARYKSYCSNISRTFLVNPTEAMQENYTFLVSVQEEILK-LLVPGTKLCDVYEKTLDFVK---KEKPSM
H.s._SPT16H     268 FG---AITCAMGIRFKSYCSNLVRTLMVDPSQEVQENYNFLLQLQEELLK-ELRHGVKICDVYNAVMDVVK---KQKPEL
M.m._Supt16     268 FG---AITCAMGIRFKSYCSNLVRTLMVDPTQEVQENYNFLLQLQEELLK-ELRHGVKICDVYNSVMDVVK---KQKPEL
```

Spt16N_Peptidase M24

```
P.t._SPT16-1    341 ---KMKFP-TDIGYLIG-----SQMLIDNHNIE-TIQDRMAVVIRMFVDNILVQLPFYPERTNIAICLADTIFV---VSG
T.t._SPT16      342 ---KDKIP-ANFGFGIGLEFRESNLYINAKNEK-EVEEGMVFNVVVGFDNLQSE-----KEKAYAIQISDTVAI-RKQNT
P.t._SPT16-2c   323 NITNVQLP-SSFGYGIGLELKEPFLAISEKSTH-LVAKNEVYFVQVTLENLSNGQ----KGISYTISVGDVIVV---TNG
P.t._SPT16-2a   323 NLQNLQLP-TSFGYGIGLELKESCLTINEKSTH-VVTKGEVYFVNVSLENVQNGQ----KNITYTVQVGDVIVI---TNG
P.t._SPT16-2b   323 NLLNLQLP-NSLGYGIGLELKESNLVINEKSTH-VVAKGEVYFVNVVMENVPNGQ----KNIVYTIQVGDVIVI---TNG
A.t._SPT16      370 ---VDKLT-KSAGTGIGLEFRESGLNINAKNDK-VLRPKMAFNVSLGFQNLECESESRSKNKKFSLLLADTVLV---TDQ
S.p._Spt16      349 ---EPNFV-RNLGAGIGIEFRESSLLVNAKNPR-VLQAGMTLNLSIGFGNLINPHPKNSQSKEYALLLIDTIQI---TRS
S.c._Spt16      357 ---VPNFT-KNIGSLIGLEFRDSNFILNVKNDYRKIQRGDCFNISFGFNNLKDSQ----SANNYALQLADTVQIPLDETE
C.e._SPT16      340 ---AETLNKKELGFATGIEFRESRLAISAKCDE-VVKAGMVFIVYIGVDSIPNKN-KGEKGKPAAIAISDTILV--KEEG
D.m._Dre4       342 ---VDNLP-KSFGFAMGLEFRENSIVIGPKCQA-LLKKNMVFNLHVGISNLTNPEATDKEGKNYALFIGDTVLV--GEQS
H.s._SPT16H     341 ---LNKIT-KNLGFGMGIEFREGSLVINSKNQY-KLKKGMVFSINLGFSDLTNKEGKKPEEKTYALFIGDTVLV--DEDG
M.m._Supt16     341 ---LNKIT-KNLGFGMGIEFREGSLVINSKNQY-KLKKGMVFSINLGFSDLTNKEGKKPEEKTYALFIGDTVLV--DEDG
```

Spt16N_Peptidase M24

```
P.t._SPT16-1    408 IEDCVITKAEKEFTFVSYQPTEEGE----------RFFK--STFQKNENSD-VLHQSEKITR----EQFEQAELNKIKND
T.t._SPT16      411 PNAVMTFKVSKKYEDISYSIQDEGQDEEQEEEEDDLEKE--NIIQDGRRTRNAYHKNTTIVS---------EKERQKH--
P.t._SPT16-2c   394 AANVTTNSIPKAYKQISYQLQEEDEQPAQKQ----QPKQ--TDAPKEGRTRAPRNQQIQIQR-------ENEKQRQIH--
P.t._SPT16-2a   394 ATTITTQQIPKAYKQISYQLQEEDEPERKP-----APVQ--TDKDKPIRAR-PRNQQIQIQR-------ENEKQRQIH--
P.t._SPT16-2b   394 AATITTQQIPKAYKQISYQLQEEDEPERKP-----APVQ--TDKDKPIRAR-PRNQQIQIQR-------ENEKQRQIH--
A.t._SPT16      442 KPELLTK-CSKSVKDVAYSFKEDEEEE--------KPRK--KARTSGSENY-ITKTALRSDD----HVVSKEELRKQH--
S.p._Spt16      421 DPIVFTD-SPKAQGDISYFFGEDDSSLEDGV----KPRK---PPTRGTATISSHKGKTRSET--RDLDDSAEKRRVEH--
S.c._Spt16      429 PPRFLTN-YTKAKSQISFYFNNEEEDNNKKKS---SPAT--KVPSKPDRNSKILRTKLRGEARGGAEDAQKEQIRKEN--
C.e._SPT16      413 DNEILTEKAKSRLKSNVIKFKEEQENREAEKD---NDQK--KMLGRGQRSV-VLTDQTRNKT-------TNEELRKER--
D.m._Dre4       415 PASVMTP-SKKKIKNVGIFIKDDSDEEDVDDKKTAKEDQGTEILGRSKRNA-VLESKLRNEI-------NTEEKRKEH--
H.s._SPT16H     414 PATVLTS-VKKKVKNVGIFLKNEDEEEEEEK---DEAE--DLLGRGSRAA-LLTERTRNEM-------TAEEKRRAH--
M.m._Supt16     414 PATILTS-VKKKVKNVGIFLKNEDDEEEEEK---DEAE--DLLGRGSRAA-LLTERTRNEM-------TAEEKRRAH--
```

Spt16D

```
P.t._SPT16-1    471 QEKLKEIKQYELEVRLNDQQ--TRQEPKLLVKMDQLQAFQKEDQFDQ---YPKGEIAVDQDKSAILIPIIGTHYPFHALT
T.t._SPT16      478 QLELREVKLKELQERYNNNGFLSNKINSRALELDKVQCYGGPQDIPKE--YKKNQIHIDAAHNAILLPVNGELVPFHISL
P.t._SPT16-2c   459 QEKLAKDKQNELEQRLEQDQFVQNSQETKALELDKLQCYQKSEQYPKD--LQKGQIYIDNQKCAVLVPLLGTHVPFHVSC
P.t._SPT16-2a   457 QEKLAKDKQTELEQRLEQDQFVQNQQEVKALELDKLLCYQRPEQYPKE--LQKGQIYIDNQKCALLVPLMGTHIPFHVSC
P.t._SPT16-2b   457 QEKLAKDKQTELEQRLEQDQFVQNQQEVKALELDKLLCYQRPEQYPKE--LQKGQIYIDNQKCALLVPLMGTHIPFHVSC
A.t._SPT16      504 QAELARQKNEETARRLAGDSSGAGDSRSTAKTSADVVAYKNVNDMPHK----ELMIQVDTRNEAVLLPIYGSLVPFHVAT
S.p._Spt16      489 QKQLASRKQAEGLQRFAQGSVPSSGIEKP--TVKRFESYKRDSQLPQA--IGELRILVDYRAQSIILPIFGRPVPFHIST
S.c._Spt16      501 QKKLHEKLEKNGLLRFSAADA-NGPDSEPRQYFKKYESYVRDSQLPTN--IRDLRIHVDWKSQTIILPIYGRPVPFHINS
C.e._SPT16      478 QKELGVQLNELAKARLSKQGGGTDEKKSK----KSNVSYKTEERFPQDADVQKMLIFVDRKYDSVVVPIFGIPVPFHISM
D.m._Dre4       484 QRELAQQLNERAKDRLARQGNSKEVEKVR----KNTVSYKSISQMPREPEVKELKLYVDKKYETVIMPVFGIQVPFHIST
H.s._SPT16H     478 QKELAAQLNEEAKRRLTEQKGEQQIQKAR----KSNVSYKNPSLMPKEPHIREMKIYIDKKYETVIMPVFGIATPFHIAT
M.m._Supt16     478 QKELAAQLNEEAKRRLTEQKGEQQIQKAR----KSNVSYKNPSLMPKEPHIREMKIYIDKKYETVIMPVFGIATPFHIAT
```

Spt16D

```
P.t._SPT16-1   546 IQNVSVKELPNGAGEITIRFWTNEFHID---TR-EFPSM-DQDQMFLKEITLRNQEFIK--------LQDIENEINVCRD
T.t._SPT16     556 IKNYSKNDEGKT-HTLRLNFHNPGSGSN-LANI-TFPKI-DGQIVFIKELTFRSKN-----------AKNMLETIKKIKD
P.t._SPT16-2c  537 IKNMG--------SSIRINFFTSETTAG---QI-QFPQI-DGETIFIKELQYRSKK-----------SDRPQNLILQIKQ
P.t._SPT16-2a  535 IKNVSKIDEGKMGSSIRINFFTSETTAG---QI-QFPKV-DGETIFIKELQYRSKK-----------SDRPQNLILQIKS
P.t._SPT16-2b  535 IKNVSKIDEGKMGSSIRINFFTSETTAG---QI-QFPKV-DGETIFIKELQYRSKK-----------SDRPQNLILQIKS
A.t._SPT16     580 IRTVSGNQDTNRNCYIRIIFNVPGTPFNPHDSN-SLKNQ-GAI--YLKEVSFRTKD-----------SRHSSEVTQQIKT
S.p._Spt16     565 LKNASKNDEGNF-VYLRLNFVSPGQIGGKKDEL-PFEDP-NAQ--FIRSFTFRSSN-----------NSRMSQVFKDIQD
S.c._Spt16     578 YKNGSKNEEGEY-TYLRLNFNSPGSSGGISKKVEELPYEESADNQFVRSITLRSKD-----------GDRMSETFKQIAD
C.e._SPT16     554 IKNCSQSVEGDF-TYLRINFATPGSQVG-KDSG-QFPHP-LAH--YMKELTFRASNIKDHHSDSTAPSHNLSTAFRLIKE
D.m._Dre4      560 IKNISQSVEGEY-TYLRINFFHPGATMGRNEGG-LYPQP-EAT--FVKEVTYRSSNVKE-HGEVGAPSANLNNAFRLIKE
H.s._SPT16H    554 IKNISMSVEGDY-TYLRINFYCPGSALGRNEGN-IFPNP-EAT--FVKEITYRASNIKA-PGEQTVPALNLQNAFRIIKE
M.m._Supt16    554 IKNISMSVEGDY-TYLRINFYCPGSALGRNEGN-IFPNP-EAT--FVKEITYRASNMKA-PGEQTVPALNLQNAFRIIKE
```

```
P.t._SPT16-1   613 DARRKQIEKQLEVDKFDFVIEKLTVLPKN--TP-CLSKVYMRPTQSQKTRSPEGFVECHENGFRYKS-ARGE-VIDFTFT
T.t._SPT16     621 LQAKVKQTDQEAKNKDELVEQDKLQL-RNTKRP-ALRNLKVRPAISK-QKVN-GMLELHLNGFRYMT-TKNE-KVDVIFK
P.t._SPT16-2c  593 LQKKVKAEQQVEREKQHVGELEPLIVSKSGRKP-IFKDLKVRPTFGS-GKAA-GILEVNSNGFRYVH-SNKE-QLDIVFK
P.t._SPT16-2a  599 LQKKVKTEQQVEREKQNVGEMEPLIINKGGRKP-IFKDLKVRPTFGS-GKAA-GILEVHTNGFRYIH-SNKE-QLDIVFK
P.t._SPT16-2b  599 LQKKVKTEQQVEREKQNVGETEPLIINKGGRKP-IFKDLKVRPTFGS-GKAA-GILEVHTNGFRYIH-SNKE-QLDIVFK
A.t._SPT16     645 LRRQVMARESERAERATLVTQEKLQLAGNKFKPLRLSELWIRPPFSGRKKIP-GTLEAHANGFRYST-TRPDERVDVLFA
S.p._Spt16     629 MKKAATKRETERKEFADVIEQDKLIEIKNKRPA-HINDVYVRPAIDG-KRLP-GFIEIHQNGIRYQSPLRSDSHIDLLFS
S.c._Spt16     646 LKKEATKREQERKALADVVQQDKLIENKTGRTK-RLDQIFVRPNPDT-KRVP-STVFIHENGIRFQSPLRTDSRIDILFS
C.e._SPT16     628 MQKRFKTEEAEEREKEGAVKQDKLILSQNKLNP-KLKDLLIRPNIIQ-KRIT-GSLEAHTNGFRYTS-LRGD-RIDVLYN
D.m._Dre4      634 VQKRFKTREAEEREKEDLVKQDTLILSQNKGNP-KLKDLYIRPNIVT-KRMT-GSLEAHSNGFRYIS-VRGD-KVDILYN
H.s._SPT16H    628 VQKRYKTREAEEKEKEGIVKQDSLVINLNRSNP-KLKDLYIRPNIAQ-KRMQ-GSLEAHVNGFRFTS-VRGD-KVDILYN
M.m._Supt16    628 VQKRYKTREAEEKEKEGIVKQDSLVINLNRSNP-KLKDLYIRPNIAQ-KRMQ-GSLEAHVNGFRFTS-VRGD-KVDILYN
```

```
P.t._SPT16-1   688 SIKHCFFVSPEDEVIACIHFIFKMPIKCGKIMFSQIQFYRDIEGASEQEAAR------RKVRLFDIDHVF-DKKVQDRRL
T.t._SPT16     695 NIKHAIFQPCDNEMIVAIHFNLKNPIMIGKKKVWDVQFYTEAGLPPEDLN--------NRRRGHDYDEIEEEQMEKARRK
P.t._SPT16-2c  668 NIKHYIYQSPEQDIIAALHFHLHSPIVLGKRKTHDVQFYCEVGGAVEHLEGR------RKNNKNDDDEIEEEERLRVHRK
P.t._SPT16-2a  674 NIKHYIYQSPEQDIIAALHFHLHSPIVLGKRKTHDVQFYCEVGGAVEHLEGR------KKTNRNDEDEIEEEERLRMHRR
P.t._SPT16-2b  674 NIKHYIYQSPEQDIIAALHFHLHSPIVLGKRKTHDVQFYCEVGGAVEHLEGR------KKTNRNDEDEIEEEERLRMHRR
A.t._SPT16     723 NIKHAFFQPAEKEMITLLHFHLHNHIMVGTKKTKDVQFYVEVMDVVQSLGG-------GRRSAYDPDEIDEEQRERDRKN
S.p._Spt16     706 NMKHLFFQPCEGELIVLIHVHLKAPIMVGKRKTQDVQFYREVSDIQFDETGNKK----RKYMYGDEDELEQEQEERRRRA
S.c._Spt16     723 NIKNLIFQSCKGELIVVIHIHLKNPILMGKKKIQDVQFYREASDMSVDETGGGRRGQSRFRRYGDEDELEQEQEERRKRA
C.e._SPT16     703 NIKHAFFQPCDNEMIILLHFHLKNPVLWGKKKYKDVQFYTEVGEITTDLG--------KYHHMQDRDDMQSEQQEREMRR
D.m._Dre4      709 NIKSAFFQPCDGEMIILLHFHLKYAIMFGKKKHVDVQFYTEVGEITTDLG--------KHQHMHDRDDLAAEQAERELRH
H.s._SPT16H    703 NIKHALFQPCDGEMIIVLHFHLKNAIMFGKKRHTDVQFYTEVGEITTDLG--------KHQHMHDRDDLYAEQMEREMRH
M.m._Supt16    703 NIKHALFQPCDGEMIIVLHFHLKNAVMFGKKRHTDVQFYTEVGEITTDLG--------KHQHMHDRDDLYAEQMEREMRH
```

Spt16M_Rtt106

```
P.t._SPT16-1   761 EELKNFESFIQQSEQY-----YKRFNIKFERLEKQYSFEGNYAKERVVFQPTQSCLVNIVDQPFFTLTLENVDIMCCERV
T.t._SPT16     767 KLNKDFEAFYKEVENQ------LGDKIKFEVPYANLGFYGSPSRSTCLLQPTQNTLMNIIEFPFFIMSLEEVELACFERM
P.t._SPT16-2c  742 KMAREFEVFIKTIEEM-----GADYKIQFEKPFRDLGFEGNWNRARLFLQPTHNTLMNVVESPFFILTLSEVEICCFERI
P.t._SPT16-2a  748 KMAKEFEVFIKTIEEL-----GADYKISFEKPFRDLGFEGNWNRARLFLQPTRDTLMNVVESPFFILTLNEVEICCFERI
P.t._SPT16-2b  748 KMAKEFEVFIKTIEEL-----GADYKISFEKPFRDLGFEGNWNRARLFLQPTRDTLMNVVESPFFILTLNEVEICCFERI
A.t._SPT16     796 KINMDFNHFANRVNDMWQLPQFASLDLEFDQPLRELGFHGVPHKTSAFIIPTSSCLVELIEYPFLVVSLSEIEIVNLERV
S.p._Spt16     782 QLDREFKSFAEKIAEA------SEGRIELDIPFRELAFNGVPFRSNVLLQPTTDCLVQLTDTPFTVITLNEIEIAHLERV
S.c._Spt16     803 ALDKEFKYFADAIAEA------SNGLLTVENTFRDLGFQGVPNRSAVFCMPTTDCLVQLIEPPFLVINLEEVEICILERV
C.e._SPT16     775 RLNAAFNSFCEKVSRL------TNDQFEFDSPFAGLGFFGVPYRSATTLKPTASCLVNLTEWPTFIVTLSEVELVHFERV
D.m._Dre4      781 KLKTAFKSFCEKVETM------TKSVVEFDTPFRELGFPGAPFRSTVTLQPTSGSLVNLTEWPPFVITLDDVELVHFERV
H.s._SPT16H    775 KLKTAFKNFIEKVEAL------TKEELEFEVPFRDLGFNGAPYRSTCLLQPTSSALVNATEWPPFVVTLDEVELIHFERV
M.m._Supt16    775 KLKTAFKNFIEKVEAL------TKEELEFEVPFRDLGFNGAPYRSTCLLQPTSSALVNATEWPPFVVTLDEVELIHFERV
```

```
                             Spt16M_Rtt106
P.t._SPT16-1    836 QEETISFDLVAVLKDLEAQVIRIEAIDREDLKKIQQWLNKKKILFFQTTSGLMWRNMQFSIQKDFPLFVYDGGWATMMKD
T.t._SPT16      841 IGRLKNFDLVFIFKDYEKQVTRIASIPIDKAEIVKNWLNSQNILYFESTKSFSWANILKTIRQDIGGFIEDGGWNIILG-
P.t._SPT16-2c   817 IPGIKSFDLVFVFKNYDRQVLRIESIDIKDLEGVKNWLDRMNILFFEVAQNLVWKNVLAQIQKDIPGFVQDGGWTNILAE
P.t._SPT16-2a   823 IPGIKSFDLVFVFKNYDKQVLRIESIDIKDLEGVKNWLDRMNILFFEVGQNLVWKNVLGQIQKDIPGFVQDGGWTNILAE
P.t._SPT16-2b   823 IPGIKSFDLVFVFKNYDKQVLRIESIDIKDLEGVKNWLDRMNILFFEVGQNLVWKNVLGQIQKDIPGFVQDGGWTNILAE
A.t._SPT16      876 GFGQKNFDMAIIFKDFKKDVLRVDSVPTSSLEGIKEWLDTTDIKYYESKLNLNWRQILKTITDDPQSFIDDGGWEFLNLD
S.p._Spt16      856 QFGLKNFDLVFIFQDFRRPPIHINTIPMEQLDNVKEWLDSCDICFYEGPLNLNWTTIMKTVNEDPIAFFEEGGWGFLGAP
S.c._Spt16      877 QFGLKNFDMVFVYKDFNKPVTHINTVPIESLDFLKQWLTDMDIPYTVSTINLNWATIMKSLQDDPYQFFLDGGWNFLATG
C.e._SPT16      849 SLQLKNFDMVFIFKDYKIKPQMVAQIPMSSIDKIKEWLHTCDIWYSEGIQSLNWAKVMKTITDDLEAFFEEGGWSFLNVE
D.m._Dre4       855 QFHLRNFDMIFVFKEYNKKVAMVNAIPMNMLDHVKEWLNSCDIRYSEGVQSLNWQKIMKTITDDPEGFFEQGGWTFLDPE
H.s._SPT16H     849 QFHLKNFDMVIVYKDYSKKVTMINAIPVASLDPIKEWLNSCDLKYTEGVQSLNWTKIMKTIVDDPEGFFEQGGWSFLEPE
M.m._Supt16     849 QFHLKNFDMVIVYKDYSKKVTMINAIPVASLDPIKEWLNSCDLKYTEGVQSLNWTKIMKTIVDDPEGFFEQGGWSFLEPE

                               ** *     Spt16C_MBD
P.t._SPT16-1    916 HM--EHAPIQQFNDEP--LEEPDSSNGPTSVSEFEFEQDKKNNKYLHLQKDDESDFSDLVDSE--DIMSELDIQERRKRK
T.t._SPT16      920 ----EDEGEDEDPDEGDEDFSVEQDE--------EEEESEEISESDEDFDDEESASDFEGDEDLSEEGQSWSDLEENARK
P.t._SPT16-2c   897 SE--EEEGDEDDPEAGDSEFSPEQSEE-------DGDDDSDFIEDDDDGDDEDDDDGGSDDDDL-SDVLDLNNISEQDDD
P.t._SPT16-2a   903 SE--EEGDEEDDPEAGDSEFSPGESGD-------DGDDDSDFIEDDEDGDDDGDDDD-DDEDL--SDVLDLNKISDQDED
P.t._SPT16-2b   903 SE--EEGDEEDDPEAGDSEFSPGESGD-------DGDDDSDFIEDDEDGDDDDGDDDDDDEDL--SDVLDLNKISDQDED
A.t._SPT16      956 GS--DSESGGSEESDK--GYEPSDVEVES-----ESEDEASESESLVESDDDEEEDSEQESEE--EKGKTWDELEREATN
S.p._Spt16      936 S---DDEGDDSVEEVS--EYEASDADP-------SDEEEEESEEYSEDASEEDGYSESEVEDE--ESGEDWDELERKARQ
S.c._Spt16      957 SD--DEASDESEEEVS--EYEASEDDVSD---ESAFSEDEEGSEVDDDISGDESEDYT-GDES--EEGEDWDELEKKAAR
C.e._SPT16      929 SD--NEEAMDDSDDSD--AYDPEEEDASAGSGS-ESDEDESEGE---ETESDDDDEGSLDSDE--SEGKDWSDLEEEAAN
D.m._Dre4       935 SG--SEGENETAESEEDEAYNPTDAE--------SDEESDEDSEYSEASEDSEESDEDLGSDE--ESGKDWSDLEREAAE
H.s._SPT16H     929 GEGSDAEEGDSESEIEDETFNPSEDDY-------EEEEEDSDEDYSSEAEESDYSKESLGSEE--ESGKDWDELEEEARK
M.m._Supt16     929 GEGSDAEDGDSESEIEDETFNPSEDDY-------EEEEEDSDEDYSSEAEESDYSKESLGSEE--ESGKDWDELEEEARK

P.t._SPT16-1    990 K--VKYNE-------------ID---------------------------------------------------------
T.t._SPT16      988 EEERR-----------------------------KQQQVKPTKPV---------------PQKRR---------------
P.t._SPT16-2c   967 DD-DESDS-------------DKPRHKNKSKPQSKPQPKPQQKPTSVKPTQ----QQKRPPRK-----------------
P.t._SPT16-2a   971 DEDEDSDS-------------DKNRRKANSRPQQKPQHKPQNKPQGKPQQS-VQPHRSNQPQKRQPP-------------
P.t._SPT16-2b   972 DEDEDSDS-------------DKHKRKANSRPQQKPQHKPQNKPQQHKPQQSGPPPRSNQPQKRQPPRK-----------
A.t._SPT16     1025 AD-REHGV-------------ESDSEEERKRR--KMKAFGKSRPGTSGGGG-SSSMKNMPPSKRKHR-------------
S.p._Spt16     1002 EDAKHDAE-------------EE------------------------------------RPSKKRHR-------------
S.c._Spt16     1027 AD-RGANE-------------RD---------------------------------------------------------
C.e._SPT16      999 AD-KRREV-------------EE-----------PSRDRDRKRPHSSKSG----------PSHKRRK-------------
D.m._Dre4      1003 ED-RNHDYAADDKPRNGKFDSKKHGKSSKHSPSKSSKDKYNSRDKHHSSSSSGNKSSSKDKDRKRSRDDSRDNGHKSKKS
H.s._SPT16H    1000 AD-RESRY-------------EEEEEQSR-----SMSRKRKASVHSSGRGSNRGSRHSSAPPKKKRK-------------
M.m._Supt16    1000 AD-RESRY-------------EEEEEQSR-----SMSRKRKASVHSSGRGSNRGSRHSSAPPKKKRK-------------

P.t._SPT16-1        --
T.t._SPT16          --
P.t._SPT16-2c       --
P.t._SPT16-2a       --
P.t._SPT16-2b       --
A.t._SPT16          --
S.p._Spt16          --
S.c._Spt16          --
C.e._SPT16          --
D.m._Dre4      1082 RH
H.s._SPT16H         --
M.m._Supt16         --
```
