# Supplementary material for: The Paramecium histone chaperone Spt16-1 is required for Pgm endonuclease function in programmed genome rearrangements
Source: PLoS Genet. 2020 Jul 23;16(7):e1008949. doi: 10.1371/journal.pgen.1008949 (PMC7402521; doi:10.1371/journal.pgen.1008949)
Supplement: S1 File — Related to legends of Fig 1, Fig 2 and Fig 3. (DOCX) [file pgen.1008949.s010.docx]

**S1 File. Related to legends of Fig 1, Fig 2 and Fig 3.**

**Related to Fig 1.** RNA sequencing was performed during vegetative growth and at different time points during autogamy [1]. The vegetative point consists of two samples from mass cultures containing only log-phase cells showing no sign of meiosis. The meiosis time point consists of two samples containing 32-39% of cells undergoing meiosis and little fragmentation of the maternal MAC. The fragmentated time point consists of three samples containing a similar proportion of meiotic cells (20-23%) as the meiosis time point, but also contained 40-47% of cells with a fragmented maternal MAC. The Development 1 time point consists of two samples containing 50-56% of cells with a fragmented maternal MAC and 35-37% of cells that contained two already visible new MACs. The development 2/3 time point consists of four samples containing 73-91% of cells with two new MACs. Development 4 time point consists of two samples taken 10 hours after the previous time point and consisting of 91-98% of cells with two new MACs and in which appear 1-9% of post-autogamous cells.

**Related to Fig 2.**

Cells were starved in each medium to induce autogamy and, following 3-4 days of starvation, autogamous cells were transferred individually to *K. pneumoniae* medium to monitor growth of sexual progeny. Reactive cells from opposing mating types were crossed in standard *K. pneumoniae* medium, and mating pairs were transferred to silencing medium to allow pre-zygotic events, including karyogamy and mitoses of the zygotic nucleus, to occur before cells began to ingest dsRNA from bacteria, once the conjugating pairs separated. Conjugating pairs separate, coinciding with the onset of MAC development [2]. The following day cells were transferred to standard food medium to resume vegetative growth.

**Related to Fig 3.**

Transgene copy number per haploid genome was 18 as measured by qPCR from total genomic DNA extracted from vegetative cells and values were normalized to the endogenous *GAPDH* gene. Cells were starved in each medium to induce autogamy and, following 3-4 days of starvation, autogamous cells were transferred individually to *K. pneumoniae* medium to monitor growth of sexual progeny.

**References**

1. Arnaiz O, Dijk EV, Bétermier M, Lhuillier-Akakpo M, Vanssay A de, Duharcourt S, et al. Improved methods and resources for paramecium genomics: transcription units, gene annotation and gene expression. BMC Genomics. 2017;18: 483. doi:10.1186/s12864-017-3887-z

2. Berger JD. Nuclear differentiation and nucleic acid synthesis in well-fed exconjugants of Paramecium aurelia. Chromosoma. 1973;42: 247–68.
