# Supplementary material for: The Paramecium histone chaperone Spt16-1 is required for Pgm endonuclease function in programmed genome rearrangements
Source: PLoS Genet. 2020 Jul 23;16(7):e1008949. doi: 10.1371/journal.pgen.1008949 (PMC7402521; doi:10.1371/journal.pgen.1008949)
Supplement: S1 Table — Accession number, name of the protein and species are given. Paramecium database: https://paramecium.i2bc.paris-saclay.fr/, T. thermophila database: http://ciliate.org/index.php/home/welcome, S. pombe database: https://www.pombase.org/, S. cerevisiae database: https://www.yeastgenome.org/, C. elegans database: https://wormbase.org/#012-34-5, D. melanogaster database: http://flybase.org/, M. musculus database: http://www.informatics.jax.org/, H. sapiens database: https://www.genenames.org, A. thaliana database: https://www.arabidopsis.org/. (DOCX) [file pgen.1008949.s012.docx]

| Species | Name | Accession Number |
| --- | --- | --- |
| *Paramecium tetraurelia* | Spt16-1 | PTET.51.1.P0710091 |
| *Paramecium tetraurelia* | Spt16-2a | PTET.51.1.P1210069 |
| *Paramecium tetraurelia* | Spt16-2b | PTET.51.1.P1050068 |
| *Paramecium tetraurelia* | Spt16-2c | PTET.51.1.P0550178 |
| *Tetrahymena thermophila* | Spt16 | TTHERM_00283330 |
| *Schizosaccharomyces pombe* | Spt16 | SPBP8B7.19 |
| *Saccharomyces cerevisiae* | Spt16 | YGL207W |
| *Caenorhabditis elegans* | Spt16 | WP:CE17113 |
| *Drosophila melanogaster* | DRE4 | FBgn0002183 |
| *Homo sapiens* | Spt16H | HGNC:11465 |
| *Mus musculus* | Supt16 | MGI:1890948 |
| *Arabidopsis thaliana* | Spt16 | AT4G10710 |
| *Paramecium primaurelia* | Spt16-1 | PPRIM.AZ9-3.1.P1650085(*) |
| *Paramecium primaurelia* | Spt16-2a | PPRIM.AZ9-3.1.P0700122 |
| *Paramecium primaurelia* | Spt16-2b | PPRIM.AZ9-3.1.P0670199 |
| *Paramecium primaurelia* | Spt16-2c | PPRIM.AZ9-3.1.P1030093 |
| *Paramecium primaurelia* | Spt16-2d | PPRIM.AZ9-3.1.P0690041 |
| *Paramecium biaurelia* | Spt16-1 | PBIA.V1_4.1.P01200073 |
| *Paramecium biaurelia* | Spt16-2a | PBIA.V1_4.1.P00180125 |
| *Paramecium biaurelia* | Spt16-2b | PBIA.V1_4.1.P00690070 |
| *Paramecium biaurelia* | Spt16-2c | PBIA.V1_4.1.P04930005 |
| *Paramecium biaurelia* | Spt16-2d | PBIA.V1_4.1.P02300049 |
| *Paramecium pentaurelia* | Spt16-1 | PPENT.87.1.P0520132 |
| *Paramecium pentaurelia* | Spt16-2a | PPENT.87.1.P0610195 |
| *Paramecium pentaurelia* | Spt16-2b | PPENT.87.1.P0440198 |
| *Paramecium pentaurelia* | Spt16-2c | PPENT.87.1.P0970092 |
| *Paramecium pentaurelia* | Spt16-2d | PPENT.87.1.P0510097 |
| *Paramecium sexaurelia* | Spt16-1 | PSEX.AZ8_4.1.P1620011 |
| *Paramecium sexaurelia* | Spt16-2a | PSEX.AZ8_4.1.P0570148 |
| *Paramecium sexaurelia* | Spt16-2b | PSEX.AZ8_4.1.P0960067 |
| *Paramecium sexaurelia* | Spt16-2c | PSEX.AZ8_4.1.P1020092 |
| *Paramecium sexaurelia* | Spt16-2d | PSEX.AZ8_4.1.P0830089 |
| *Paramecium octaurelia* | Spt16-1 | POCTA.138.1.P1140149 |
| *Paramecium octaurelia* | Spt16-2a | POCTA.138.1.P1350085 |
| *Paramecium octaurelia* | Spt16-2b | POCTA.138.1.P1280104 |
| *Paramecium octaurelia* | Spt16-2c | POCTA.138.1.P1200107 |
| *Paramecium tredecaurelia* | Spt16-1 | PTRED.209.2.P71800001294160129 |
| *Paramecium tredecaurelia* | Spt16-2a | PTRED.209.2.P71800001294140062 |
| *Paramecium tredecaurelia* | Spt16-2b | PTRED.209.2.P71800001294090202 |
| *Paramecium tredecaurelia* | Spt16-2c | PTRED.209.2.P71800001290980080 |
| *Paramecium tredecaurelia* | Spt16-2d | PTRED.209.2.P71800001293940093 |
| *Paramecium sonneborni* | Spt16-1 | PSON.ATCC_30995.1.P0740129 |
| *Paramecium sonneborni* | Spt16-2a | PSON.ATCC_30995.1.P1010097 |
| *Paramecium sonneborni* | Spt16-2b | PSON.ATCC_30995.1.P1090084 |
| *Paramecium sonneborni* | Spt16-2c | PSON.ATCC_30995.1.P1030100 |
| *Paramecium sonneborni* | Spt16-2d | PSON.ATCC_30995.1.P1130112 |
| *Paramecium caudatum* | Spt16-1 | PCAU.43c3d.1.P00040086 |
| *Paramecium caudatum* | Spt16-2 | PCAU.43c3d.1.P00700076 |

| Species | Name | Accession Number |
| --- | --- | --- |
| *Paramecium tetraurelia* | Pob3-1 | PTET.51.1.G0610231 |
| *Paramecium tetraurelia* | Pob3-2a | PTET.51.1.G0340178 |
| *Paramecium tetraurelia* | Pob3-2b | PTET.51.1.G0610125 |
| *Tetrahymena thermophila* | Pob3 | TTHERM_00049080 |
| *Schizosaccharomyces pombe* | Pob3 | O94529 |
| *Saccharomyces cerevisiae* | SSRP1 | Q04636 |
| *Caenorhabditis elegans* | SSRP1 | O01683 |
| *Drosophila melanogaster* | SSRP1 | Q05344 |
| *Homo sapiens* | SSRP1 | Q08945 |
| *Arabidopsis thaliana* | SSRP1 | Q05153 |

**S1 Table.** **Accession number of proteins used in the phylogenetic analyses.**

Accession number, name of the protein and species are given. *Paramecium* database: https://paramecium.i2bc.paris-saclay.fr/, *T. thermophila* database: http://ciliate.org/index.php/home/welcome, *S. pombe* database: https://www.pombase.org/, *S. cerevisiae* database: https://www.yeastgenome.org/, *C. elegans* database: https://wormbase.org/#012-34-5, *D. melanogaster* database: http://flybase.org/, *M. musculus* database: http://www.informatics.jax.org/, *H. sapiens* database: https://www.genenames.org, *A. thaliana* database: https://www.arabidopsis.org/.

* *P. primaurelia* Spt16-1 protein corrected based on alignement with *P. tetraurelia* Spt16-1

> *P. primaurelia* Spt16-1

MDKVQIALQNKFKKNNKLAITTLKKANQFDCLVVLIGTSHTSSIGIQKGFQQWYLGCELIDCILIMSTKMLCIIADEVMFQKLKHLSDIKMKTFSIYFLIKNIKKNNHQQFQFALERLRKEYPSNNYRLALNLSDGQRSPLISEFNQFIDQNHLIKVDCTSFLKELINNDNKDIFEYYNTCGKINSYYMKFMSQRIELAIKFNENTTNYSITQAVKREKSSDLNQMAIRRKFGLQGNYDILSSTVQSGGQYNVSASESTQSRLVGDVVIYSFCCQYMQSQSYCTRTLLFQPNQELEQIYRVILNVHAFALGLVKEEIQFKQIYRETQNIWETIFKDDLDMKTKFPTDIGYLIGSQMLIDNHNSEIIQDRMAVVIRMFVDNILVQLPFYPERTNIAICLADTIFVVSGIEDCVITKAEKEFTFVSYQPTEEGERFFKSTFQKTENSDVLHQSERITREQFEQAELNKIKNDQEKLKEIKQYELEVRLNDQQTRQEPKLLVKMDQLQTFQKDDQFDQYPMGEIAVDQDKSAILIPIRGTHYPFHSLTIQNVSVKELPNGSGEITIRFWTNEFNIDTREFPSMDFDQMFLKEITLRNQEFIKLKDIENEINVCRDDARRKQIEKQMEVDKFDFVIEKLTVLPKNSPCLSKVYMRPTQSSKTRSPEGFVECHENGFRYKSARGEVIDFTFTSIKHCFFVSPEDEVIACIHFIFKMPIKCGKIQFSQIQFYRDIEGASEQEAARKKVRLFDIDHVFDKKVQDRRLEELKNFESFIQQSEQYYKRFNIKFERLEKQYSFEGNYAKERVVFQPTQSCLVNIVDQPFFTLTLENVDIMCCERVQEETISFDLVAVLKDLEAQVIRIEAIDREDIKKIQQWLNKKKILFFQTTSGLMWRNMQFSIQKDFPLFVYDGGWATMMKDHMEHAPIQQFNDEPLFEPDSSNGPTSVSEFEFEQDKKNNKYLHLQKDDESDFSDLVDSEDIMSELDIQERRKRKKVKYNFID*
