# Supplementary material for: The Paramecium histone chaperone Spt16-1 is required for Pgm endonuclease function in programmed genome rearrangements
Source: PLoS Genet. 2020 Jul 23;16(7):e1008949. doi: 10.1371/journal.pgen.1008949 (PMC7402521; doi:10.1371/journal.pgen.1008949)
Supplement: S2 Table — (DOCX) [file pgen.1008949.s013.docx]

| **Name** | **Sequence (5’ to 3’)** | **Application** |
| --- | --- | --- |
| 51A-1835-5'(3) | GGTTGCGTAACACTTCCTCTTAAATGTGAG | PCR around IESA1835 |
| 51A-1835-3' | ATCCTAACATCCTTGAATAGTTACTGATCC | PCR around IESA1835 |
| 51G1832up | GCTATAACTCTTGAAGCTGCTTGTAATATG | PCR around IESG1832 |
| 51G1832do | TTGTCAATGAGCCATTAACAGTTGCTGGAT | PCR around IESG1832 |
| 51A-4404-5'(2) | TGGAATAGTGCTGCATCACCAGCTGCTTGC | PCR around IESA4404 |
| 51A-4404-3'(2) | CCAGTTATTGAACTGCAACTTACTGCAGTG | PCR around IESA4404 |
| 51G1413up | GAAGCTGCTTGTGTTAAGAATTCTACTGG | PCR around IESG1413 |
| 51G1413do | GCATCCAGCACTAGTTGAATTTACTGTA | PCR around IESG1413 |
| MT1 | TTCTAAGCTGATTTATTCAATTTCAACAGAAC | PCR around IES mtA |
| MT2 | TTGAAAAAAGGTCATCTCTTTCATTAAATTCC | PCR around IES mtA |
| 51G2832-6 | CAAATACTGGTGGTGCAACATCCTCAACTGC | PCR around IESG2832 |
| 51G2832-8 | GAACCATTTAATGCGCAATATCCTGCATTTCC | PCR around IESG2832 |
| 51A2591-18 | AAGTGCAACCTGTGCTGATGCTCCCGATGA | PCR around IESA2591 |
| 51A2591-20 | AGTTCCTTTGAAAGATGTGCAAGCTCCAGA | PCR around IESA2591 |
| 51A4578-5' | CACTGCAGTAAGTTGCAGTTCAATAACTGG | PCR around IESA4578 |
| 51A4578-3' | GTAGTCTTAAAATCTTAGCATGTTGTACC | PCR around IESA4578 |
| 51A4578-2 | TGGTTGTTAGTCTCAAAGAATTCTAAAGAC | PCR for IES circles |
| 514578-7bis | aaattccaaaaagttttgaatatcttttgag | PCR for IES circles |
| qPCR_GFP_F1 | CTTTTCACTGGTGTTGTTCC | qPCR to measure transgene copy number |
| qPCR_GFP_R1 | TCCATAAGTTGCATCACCTT | qPCR to measure transgene copy number |
| Actin 1-1 for | AAGGTGAAATAATTGTCATCATAATCA | qPCR to measure transgene copy number |
| Actin 1-1 rev | CAACAACTGCGGGGAAG | qPCR to measure transgene copy number |

**Table S2. Oligonucleotides used in this study**
