# Supplementary material for: The Paramecium histone chaperone Spt16-1 is required for Pgm endonuclease function in programmed genome rearrangements
Source: PLoS Genet. 2020 Jul 23;16(7):e1008949. doi: 10.1371/journal.pgen.1008949 (PMC7402521; doi:10.1371/journal.pgen.1008949)
Supplement: S3 Table — Read statistics are provided for the SPT16-1 RNAi sample sequenced for this study. (DOCX) [file pgen.1008949.s014.docx]

| **Sample** | **ENA Accession** | **Insert size (bp)** | **Read length (bp)** | | **Number of reads (M)** | | **Aligned reads on the MAC** | | **MAC coverage** | | **Aligned reads on the MIC** | | **MIC coverage** | | |
| --- | --- | --- | --- | --- | --- | --- | --- | --- | --- | --- | --- | --- | --- | --- | --- |
| Control | ERX466735 | 212 | | 74 | | 106 | | 98% (104M) | | 99% | | 99% (105M) | | 79% |  |
| *PGM* RNAi | ERA137444 | 341 | | 108 | | 128 | | 87% (112M) | | 99% | | 97% (125M) | | 96% |  |
| *SPT16-1* RNAi | ERS4282798 | 372 | | 101 | | 102 | | 90,20% (92M) | | 99% | | 99% (101M) | | 96% |  |
| MIC | SAMN05323660 | 265 | | 101 | | 92 | | 84,78% (78M) | | 99% | | 98% (91M) | | 99% |  |

**S3 Table. Sequencing and mapping statistics for DNAseq.**

Read statistics are provided for the *SPT16*-1 RNAi sample sequenced for this study.
