# Supplementary material for: The Paramecium histone chaperone Spt16-1 is required for Pgm endonuclease function in programmed genome rearrangements
Source: PLoS Genet. 2020 Jul 23;16(7):e1008949. doi: 10.1371/journal.pgen.1008949 (PMC7402521; doi:10.1371/journal.pgen.1008949)
Supplement: S4 Table — Read statistics are provided for the control RNAi and SPT16-1 RNAi samples sequenced for this study. (DOCX) [file pgen.1008949.s015.docx]

| **Stage** | **Label** | **ENA Accession** | **Number of reads** | **Number of reads mapped on MAC** | **%** | **Number of reads mapped on IES** | **%** |
| --- | --- | --- | --- | --- | --- | --- | --- |
| Very Early | Control T0  *SPT16-1* T0 | ERS4282844  ERS4282840 | 14 640 580  23 589 167 | 7 232 735  14 947 237 | 49.40%  63.36% | 640 168  744 700 | 4.37%  3.16% |
| Early | Control T10 | ERS4282845 | 21 656 218 | 4 800 906 | 22.17% | 4 460 744 | 20.60% |
|  | *SPT16-1* T12 | ERS4282841 | 29 942 789 | 14 025 294 | 46.84% | 2 011 492 | 6.72% |
| Late | Control T20 | ERS4282846 | 8 974 863 | 1 785 746 | 19.90% | 2 2167 87 | 24.70% |
|  | *SPT16-1* T25 | ERS4282842 | 24 839 145 | 9 034 900 | 36.37% | 2 422 831 | 9.75% |

**S4 Table. Description of sRNA-seq data**.

Read statistics are provided for the control RNAi and *SPT16-1* RNAi sample sequenced for this study.
