# Supplementary material for: The Paramecium histone chaperone Spt16-1 is required for Pgm endonuclease function in programmed genome rearrangements
Source: PLoS Genet. 2020 Jul 23;16(7):e1008949. doi: 10.1371/journal.pgen.1008949 (PMC7402521; doi:10.1371/journal.pgen.1008949)
Supplement: Attachment — Submitted filename: Response_to_Reviewers.docx [file pgen.1008949.s016.docx]

**Response to reviewers**

**Reviewer #1:**

In this manuscript, the authors clearly show that SPT16-1 is required for programmed genome rearrangement in Paramecium and this requirement is explained by the requirement SPT16-1 in the macronuclear (MAC) localization of Pgm, the enzyme responsible for DNA excision in programmed genome rearrangement. The data presented in this manuscript are explained clearly and convincing. However, the two following points make me reluctant to fully recommend the manuscript for publication in PLoS Genetics in the current form.

We thank the reviewer for his/her comments, interest in the study, and appreciation of the data and presentation in the manuscript.

1) It is not clear whether SPT16-1 act as a part of FACT histone chaperone in programmed genome rearrangement.
Because Spt16-1 is evolutionary diverged (Figure 1), it might no longer be a histone chaperone but acquired some special function. Indeed, in Discussion, the authors mentioned that one of the Pob3 homologs in Paramecium, which shows a similar expression pattern with Spt16-1, is not required for programmed genome rearrangement. This result may indicate that Spt16-1 acts in programmed genome rearrangement not as a histone chaperone.
Does Spt16-1 forms FACT with with Pob3/SSRP1? Does Spt16-1 show any sign of histone chaperone activity? Does the loss of Spt16-1 alter chromatin architecture (such as nucleosome density, positioning, or modifications)? Especially, it is interesting (and doable) to investigate accumulations and localizations of H3K9me and H3K27me, given the fact that FACT promotes accumulation of Swi6 at heterochromatin independently of RNAi (Lejeune et al 2007).

Does Spt16-1 forms FACT with with Pob3/SSRP1? Does Spt16-1 show any sign of histone chaperone activity?

We agree with the reviewer that we do not know whether *Paramecium* Spt16-1 forms part of the histone chaperone FACT complex. We used the functional GFP-SPT16-1 fusion protein to prepare nuclear extracts. Unfortunately, GFP-Spt16-1 remained in the insoluble fraction under all extraction conditions tested (15mM NaCl to 900 mM NaCl salt extraction), suggesting GFP-Spt16-1 is insoluble under these conditions. For this reason, we cannot – for the moment – address biochemically the partners of Spt16-1.

We looked for SRRP1/Pob3 homologs in the *Paramecium* genome and identified three *POB3* genes, as indicated in the Discussion section (now included in the Results section, and in new S3 Fig). The expression of the *POB3-1* gene is specifically upregulated during development and displays a very similar expression pattern to *SPT16-1*, suggesting that they could be acting together (new S3 Fig). However, our knockdown experiments on *POB3-1* revealed it is not required for MAC development or genome rearrangements, in contrast to *SPT16-1* (new S3 Fig).

As suggested by the reviewer, these observations might be explained by Spt16-1 and Pob3-1 not forming a FACT complex. Consistent with this hypothesis, the *Paramecium tetraurelia* Pob3 protein sequences (Pob3-1 as well as the two others) lack the N-terminal N/D domain (as shown on the multiple alignments in new S4 Fig), which is required for dimerization with Spt16 in yeast (Winkler et al., 2011).

However, we cannot exclude that Spt16-1 and Pob3-1 interact indirectly. Like the *P. tetraurelia* Pob3 proteins, the Pob3 protein in the distantly related ciliate *Tetrahymena* lacks the N-terminal N/D dimerization domain (new S4 Fig) and yet pull down experiments performed in *Tetrahymena* with the unique Spt16 protein retrieved the Pob3 protein (Ashraf et al., 2019). Whether the *Tetrahymena* Spt16 and Pob3 proteins bind to each other directly or indirectly has not been tested.

Both the *P. tetraurelia* Spt16-1 and Pob3-1 proteins likely bind to histones. The aromatic and acidic amino acids required for interaction with histones are conserved in Spt16-1, suggesting it might act as a histone chaperone (S2 Fig). The domain involved in binding histones are present in Pob3, suggesting it may also function as a histone chaperone (new S4 Fig).

In conclusion, our work provides strong evidence that Spt16-1, which displays a distinct expression pattern and a divergent amino sequence compared to the Sp16-2a and 2b proteins, has a specialized role during *P. tetraurelia* development. Further studies will be needed to determine whether Spt16-1 belongs to a heterodimer FACT complex, with Pob3-1, and acts as a histone chaperone, or acts alone, either as a histone chaperone, or in some other capacity, with some other activity (although currently we think this latter possibility is less likely).

*We now include two supplementary figures. S3 Fig includes a phylogenetic tree of P. tetraurelia Pob3 proteins, their domain organization, the gene expression profiles, as well as the effect of POB3-1 RNAi knockdown on sexual progeny production and IES excision. S4 Fig shows multiple alignments of Pob3 proteins. The data is presented in the first section of the Results section now entitled “Identification of Paramecium tetraurelia Spt16 and Pob3 homologs” and discussed in the paragraph relative to the FACT complex in the Discussion section.*

Does the loss of Spt16-1 alter chromatin architecture (such as nucleosome density, positioning, or modifications)? Especially, it is interesting (and doable) to investigate accumulations and localizations of H3K9me and H3K27me, given the fact that FACT promotes accumulation of Swi6 at heterochromatin independently of RNAi (Lejeune et al 2007).

At the time Spt16-1 acts during development, three types of nuclei co-exist in the same cell: the MIC, the fragments of the maternal MAC, and the developing MAC where Spt16-1 is localized. Studying the role of Spt16-1 in nucleosome density or positioning requires the isolation of the new developing MAC from the other nuclei. Even though we recently managed to establish a flow cytometry procedure to sort developing MACs and purify genomic DNA for Illumina sequencing (Guérin et al, 2017), it is not yet technically feasible to obtain sufficient amount of chromatin from sorted developing MACs to perform MNase-Seq or ChIP-Seq experiments. Thus, assessing nucleosome density and positioning is not possible at the moment.

We were able to examine the localization of H3Kme3 and H3K27me3 during MAC development in *SPT16-1* knockdown using indirect immunostaining experiments. Depletion of Spt16-1 did not affect the deposition of H3K27me3 and H3K9me3 in the developing MACs (new Fig 6). This suggests that the Spt16-1 protein acts downstream of H3K27me3 and H3K9me3 deposition, as reported for Pgm (Lhuillier-Akakpo et al., 2014, Frapporti et al., 2019). Altogether, our data suggest Spt16-1 acts independently of the scanRNA-directed heterochromatin pathway

We also observed that H3K27me3 and H3K9me3 signals remained diffuse as development proceeds in Spt16-1-depleted cells and no foci could be detected (new S8 Fig). Spt16-1 is thus required for H3K27me3 and H3K9me3 foci formation, as we previously published for Pgm (Lhuillier Akakpo et al., 2014). We suggest that H3K9me3-H3K27me3- nuclear bodies are likely formed as a consequence of DNA double-strand break formation.

*These results are now included in a new Results section entitled “Spt16-1 is not required for H3K9me3 and H3K27me3 deposition in the developing new macronuclei” together with two additional figures (new Fig 6 and S8 Fig). We further discussed these data in the Discussion section.*

2) It is not clear whether the requirement of SPT16-1 in programmed genome rearrangement is direct or indirect.
Although it is possible that “SPT16-1 is specifically required for programmed genome rearrangements” as the authors concluded (page 9), it is also possible that Spt16-1 has some more general function in the differentiation of the MAC, which eventually indirectly promotes genome rearrangements. For example, Spt16-1 may be required for activating the zygotic gene expressions in the MAC, and zygotic expression of some gene may be essential for the proper nuclear localization of Pgm.

We agree with the reviewer that we cannot formally exclude the possibility of an indirect, general effect of Spt16-1 on MAC differentiation. When zygotic expression begins and which genes are zygotically expressed is not known.

*Therefore, we remove the word specifically in the sentence page 9. It now reads “*SPT16-1 is required for programmed genome rearrangements”.

Are PGML genes, which are known to be required for the MAC localization of Pgm (Bischerour et al., 2018), expressed normally in the absence of Spt16-1?

Genes implicated in the DNA elimination pathway are maternally expressed. Indeed, transgenes expressing tagged fusion proteins are expressed for the maternal MAC during autogamy and allow the detection of a specific signal in the developing MAC, as observed here for GFP- Spt16-1 (Fig 3). This is also true for Pgm (Baudry et al., 2009; Dubois et al., 2017) and its recently characterized partners PgmL proteins (Bischerour et al., 2018). It is unlikely that Spt16-1 drives their expression in the maternal MAC, given that GFP-Spt16-1 is exclusively found in the zygotic new developing MAC.

We examined the mRNA expression levels of genes involved in DNA elimination upon *SPT16-1* RNAi knockdown. We have generated a figure (below) for the reviewers *only* showing gene expression levels for some of these genes. We looked at “early” expressed genes, such as EZL1, DCL2, DCL3, PTIWI01, PTIWI09 and genes with an “intermediate” expression profile, such as PGM, PGML1-2-3a/b-4a/b-5a/b, Ku70a/b, Ku80c, as defined in Arnaiz et al. 2017.

After Spt16-1 depletion, we found that the transcription of genes involved in IES excision is switched on at the expected time-point during autogamy for both (early and intermediate) categories of genes, revealing that SPT16-1 is not required to induce their expression.

These data also show that, in contrast to control cells, the levels of mRNAs of “intermediate” genes (PGM, PGML, KU) do not decrease at later time-points after Spt16-1 depletion. Similar transcriptional deregulation of “intermediate” genes was observed at late autogamy time-points in KU80c and PGM knockdowns (Marmignon et al., 2014). This aberrant transcript accumulation might be a consequence of failure to complete DNA elimination. For instance, a transcriptional activator would be expressed from an IES-containing gene in the developing MAC, and switched off as soon as IES excision has been completed. When IES excision is inhibited - upon *PGM*, *KU80c* or *SPT16-1* RNAi KD-, expression of the activator would no longer be switched off, leading to continuous expression of “intermediate” genes.

In conclusion, the Spt16-1protein is not required to activate the expression of genes involved in IES excision, including the *PGML* genes also required for correct localization of the Pgm protein.

Are there any other measurable processes of the MAC differentiation, which the authors can check if they are unaffected in SPT16-1 RNAi?

There are no established markers that would allow to assess for MAC differentiation. The normal accumulation of histone marks (new Fig 6 and S8 Fig) in the developing MAC argues in favor of normal MAC differentiation at early stages upon *SPT16-1* RNAi KD.

We also considered the volume of the developing macronucleus, which increases as development progresses. We measured the nucleus volume in control and *SPT16-1* RNAi KD (see Materials and Methods) and did not detect any major changes, as shown in S9 Fig (previoulsy Fig. S6).

*We have now added this comment in the legend of S9 Fig.*

Other minor points:

3) Page 6, line 7: Why viability of non-injected SPT16-1 RNAi cells is much higher than those in the experiments shown in Figure 2A?

The reviewer is right in noticing that the viability measured in *SPT16-1* #2 RNAi KD experiments presented in Fig 3B (16% (19/120) from 2 independent experiments) is higher than the viability measured in experiments presented in Fig 2A (6 % (16/270) from 6 independent experiments, with *SPT16-1* #2 RNAi). This is most likely explained by less efficient silencing in the two experiments of Fig 3B.

4) Because the overall retention scores are much lower in PGM RNAi than those in SPT16-1 RNAi in Figure 4D, it seems eliminations of IESs are more mildly affected in SPT16-1 RNAi than in PGM RNAi. Is this because Pgm is still active in SPT16-1 RNAi and can catalyze some IES eliminations? If so, why no IES elimination was detected in SPT16-1 RNAi in Figure 4B and C? I believe some explanations are necessary for this point.

Genomic DNA was extracted from a nuclear preparation enriched for new MACs at late autogamy stages for high-throughput sequencing. At this stage, in addition to the new developing MAC, the fragments of the old maternal MAC are still present and contribute to the extracted DNA. The quantity of fragments is variable between nuclei-enriched preparations. Therefore, absolute values of RS cannot be compared from one knockdown to another, due to variable contamination by old MAC fragments. This is clearly demonstrated in a different study where we compared the retention of IESs in *PGM* RNAi KD, with or without sorting the developing MACs by flow cytometry. The absolute RS values increased with purity of the nuclei preparation (and decrease in fragments) (Guérin et al., 2017). In the present study, the distributions of RS show that *SPT16-1* knockdown strongly inhibits IES excision genome-wide (98.6% of IESs, Fig 4D). Following statistical analysis of RS (Wilkes et al., 2016), we concluded that SPT16-1, as PGM, results in significant retention of all IESs in the new MAC.

*We have added some explanation in the legend of Fig 4 to clarify this point (“Absolute values of RS cannot be compared from one knockdown to the other, due to variable contamination by old MAC fragments”).*

5) I think, for general readers, it is difficult to understand how the authors concluded that the selection of scanRNA is normal and iesRNA is reduced in Spt16-1 RNAi from the data shown in Figure 5. I think much more explanations are necessary for how to interpret the data.

We now provide more explanations in the Results section “Spt16-1 is required for iesRNA but not scnRNA accumulation”, which we hope makes it easier for the reader to follow.

**Reviewer #2:**

In this manuscript entitled “The Paramecium histone chaperone Spt16-1 is required for Pgm endonuclease function in programmed genome rearrangements”, the authors identify four Spt16-like histone chaperone homologues encoded in the genome of the ciliate Paramecium tetraurelia. These four can be classified into two gene families. They investigate the function of the most divergent one, Spt16-1, which is expressed exclusively during sexual development when it localizes specifically to developing somatic macronuclei. RNAi knockdown of this protein results in loss in the ability of cells to produce sexual progeny and failure to execute programmed elimination of germline-limited DNA. The inhibition of this major reorganization of the developing somatic genome is associated with the failure of the PiggyMac endonuclease, which initiates DNA elimination, to accumulate in the developing nucleus. Small RNAs that are required for DNA elimination accumulate normally, but a second class of small RNAs (iesRNAs) that are associated with eliminated sequences post excision do not accumulate. These finding suggests that Spt16-1 is required to ensure that this endonuclease is able to associate with the developing genome to initiate DNA elimination. The finding that an Spt16 homolog is required for DNA elimination further implicates chromatin-based regulation in this massive genome restructuring.

Strengths of the Study:
This well-executed study reports the discovery and characterization of a novel histone chaperone and shows that it has evolved to specifically regulate programmed DNA elimination in Paramecium. The data presented are quite clear and well-controlled. The RNAi inhibition of Spt16-1 expression appears to be very penetrant, leading to strong phenotypes allowing for robust conclusions. The authors show that Spt16-1 knockdown blocks all DNA elimination, and leads to a failure of Pgm accumulation in the developing nucleus, results that show that it acts after scanRNA production but concurrently or before Pgm action. This study, along with the senior author’s previous work showing a role for histone methylation in transposon silencing and DNA elimination, further highlights a role for chromatin structure in guiding genome reorganization. It has the added novelty of showing that Spt16 related proteins can undergo specialization after gene duplication and adopt specific roles in genome regulation.

*We thank the reviewer for his/her comments, noting that the study is “*well-executed*” and that the data are “*are quite clear and well-controlled*” and the RNAi experiments lead to “*robust conclusions*” and overall is enthusiastic about the study.*

Weaknesses of the study:
Major comments:
1. Although the study was well-executed, the presentation of the manuscript, including the introduction and results were rather narrowly focused on the role of Spt16-1 in programmed DNA rearrangements in Paramecium. The discovery that a specialized Spt16 exists potentially has broad implications, but the authors left speculation almost fully to the readers’ imaginations. Given the general audience of Plos Genetics, I would like to see the implications of the study made more explicit to the readers.

We agree with the reviewer that the discovery of a specialized Spt16-1 deserves more emphasis. *We have now developed this notion in the Discussion section (first paragraph).*

“In this study, we provide evidence for the functional specialization for Spt16-1. The three paralog genes Spt16-2a, -2b and -2c arising from *Paramecium* whole genome duplications [29], are constitutively expressed during vegetative growth and at all stages of the sexual cycle, and likely encode housekeeping Spt16 proteins. In contrast, Spt16-1 has a highly specialized expression pattern and a specific role during MAC development. The functional specialization of Spt16-1 is further supported by the conservation of an orthologous *SPT16-1* paralog in all species of the *P. aurelia* group, which undergo massive programmed genome rearrangements (S1 Fig).”

The authors did discuss the relationship between Spt16 more generally in DNA repair, but the parallels in function are not explored. In the canonical case, Spt16 would work after damage, but their data suggest that it acts before breaks would be induced. Thus this reviewer found the discussion a bit superficial.

We have extended the Discussion to make it clearer that Spt16-1 may act after the introduction of DNA double stand breaks. We developed what is known about the role of a specialized Ku complex in this process. In contrast to the canonical NHEJ pathway where the Ku complex binds broken ends and recruits downstream NHEJ proteins, the specialized Ku complex involved in IES excision acts upstream the introduction of DNA cleavages, coupling the DSB and repair steps (Marmignon et al., 2014).

2. In trying to connect the role of Spt16 in DNA damage and DNA elimination, is it possible that Spt16-1 interacts with the Paramecium KU complex? Could they act together in the recruitment of Pgm to sites of DNA breaks? If they have similar phenotypes, it would be interesting to test whether Spt16-1 is part of the KU complex or helps target KU to chromatin.

We agree with the reviewer’s suggestion. We have further developed our ideas about the possibility that Spt16-1 interacts with a specialized, developmental-specific Ku complex in the Discussion section. This hypothesis is consistent with a recently published paper (Abello et al., 2020) showing that KD of the Ku80c protein compromises the nuclear anchoring of Pgm, as we observe for KD of Spt16-1.

*We propose in the Discussion section a model in which Spt16-1, in association with the heterodimer Ku70/ Ku80c, tethers the Pgm/PgmL complex to chromatin, allowing Pgm to cleave DNA.*

3. What is the status of histone H3K9 and K27 methylation upon Spt16-1 knockdown? Given that this research group previously showed that “… development specific H3K27me3 and H3K9me3 ensure specific demarcation of very short germline sequences from the adjacent somatic sequences…” (from abstract of ref. #24), it sounds like they are proposing a similar role for Spt16. By not examining the status of H3 methylation, the authors have not informed the readers of the possible processes that might be affected by loss of Spt16-1 function.

*See the response to Reviewer #1, comment 1 near the end.*

We examined the localization of H3Kme3 and H3K27me3 during MAC development in *SPT16-1* knockdown using indirect immunostaining experiments. Depletion of Spt16-1 did not affect the deposition of H3K27me3 and H3K9me3 in the developing MACs (new Fig 6). This suggests that the Spt16-1 protein acts downstream of H3K27me3 and H3K9me3 deposition, as reported for Pgm (Lhuillier-Akakpo et al., 2014, Frapporti et al., 2019). Altogether, our data suggest Spt16-1 acts downstream (or in parallel) of the scanRNA-directed heterochromatin pathway

We also observed that H3K27me3 and H3K9me3 signals remained diffuse as development proceeds in Spt16-1-depleted cells and no foci could be detected (new S8 Fig). Spt16-1 is thus required for H3K27me3 and H3K9me3 foci formation, as we previously published for Pgm (Lhuillier-Akakpo et al., 2014). We suggest that H3K9me3-H3K27me3- nuclear bodies are likely formed as a consequence of DNA double-strand break formation.

*These results are now included in a new Results section entitled “Spt16-1 is not required for H3K9me3 and H3K27me3 deposition in the developing new macronuclei” together with two additional figures (new Fig 6 and S8 Fig). We further discussed these data in the Discussion section.*

Minor comments and text modifications:
1. Line 3 of introduction, suggestion: add “subsequent” to the sentence “…heterochromatin formation and subsequent DNA excision and repair...”

Done

2. Introduction, 2nd Paragraph: Flip first and second sentences. Discussing the role of the macronucleus after introducing micronuclei, then starting again to describe germline functions disrupts the information flow.

Done

3. Bottom of page 3: Typo ”sequences” also, rewrite the sentence. Histone H3 ptm are deposited on chromatin associated with sequences to be eliminated during development. If sequences have been eliminated (past tense) then they are not there to have their associated chromatin modified.

OK. It now reads:

”scanRNAs would then guide the deposition of histone H3 post-translational modifications (H3K9me3 and H3K27me3) onto sequences to be eliminated in the developing MAC and specifically tether the Pgm elimination machinery to germline sequences.”

4. Page 5 Line 7: missing “growth” “…during vegetative growth (Figure 1C)….”

Corrected

5. Page 6 Line 5: the statement: “… as no lethality was observed in the post-autogamous progeny of injected cells…” is a bit stronger than the data show. The author’s saw nearly full rescue of the RNAi-induced lethality, but it is still a bit lower than wild-type. It is accurately described lower down in the paragraph. The authors should modify the text to reflect the data.

We rephrased the sentence. it now reads:

“The GFP-Spt16-1 fusion protein was functional, as it was able to rescue lethality in the post-autogamous progeny of injected cells, following an *in vivo* complementation assay”.

6. In a few places, the authors used the word “if” when then should have used “whether”. “If” should be used in sentences that have the grammatical structure: if this, then that. When the sentence starts: “…to determine..” use “whether”, which means “to determine whether or not” something is correct.

Corrected.

1st sentence of Results section “Spt16-1 is essential for the correct nuclear localization of Pgm”

1st sentence of Results section “Identification of *Paramecium* *tetraurelia* Spt16 homologs”

7. Page 8 paragraph 2 line 5: typo, “it” not “its”

Corrected

**Have all data underlying the figures and results presented in the manuscript been provided?**
Large-scale datasets should be made available via a public repository as described in the *PLOS Genetics* [data availability policy](http://journals.plos.org/plosgenetics/s/data-availability), and numerical data that underlies graphs or summary statistics should be provided in spreadsheet form as supporting information.

Reviewer #1: Yes

Reviewer #2: Yes

PLOS authors have the option to publish the peer review history of their article ([what does this mean?](https://journals.plos.org/plosgenetics/s/editorial-and-peer-review-process#loc-peer-review-history)). If published, this will include your full peer review and any attached files.


If you choose “no”, your identity will remain anonymous but your review may still be made public.


**Do you want your identity to be public for this peer review?** For information about this choice, including consent withdrawal, please see our [Privacy Policy](https://www.plos.org/privacy-policy).

Reviewer #1: No

Reviewer #2: No
